# Targeted next-generation sequencing of 21 candidate genes in hereditary ovarian cancer patients from the Republic of Bashkortostan

**DOI:** 10.1186/s13048-023-01119-z

**Published:** 2023-04-04

**Authors:** D. S. Prokofyeva, E. T. Mingazheva, Ya. V. Valova, D. D. Sakaeva, R. R. Faishanova, A. Kh. Nurgalieva, R. R. Valiev, N. Bogdanova, T. Dörk, E. K. Khusnutdinova

**Affiliations:** 1 Federal State Budgetary Educational Institution of Higher Education, Ufa University of Science and Technology, Ufa, 450076 Russia; 2 Institute of Biochemistry and Genetics, Ufa Federal Research Center of the Russian Academy of Sciences, Ufa, 450054 Russia; 3 Ministry of Health of the Republic of Bashkortostan State Autonomous Healthcare Institution, Republican Clinical Oncology Center, Ufa, 450008 Russia; 4 Department of Obstetrics and Gynecology, Hannover Medical School, 30625 Hannover, Germany

**Keywords:** Hereditary ovarian cancer, Target sequencing, Germline mutations, Pathogenic variants, Likely pathogenic variants

## Abstract

About 5–10% of all ovarian cancer cases show familial clustering, and some 15–25% of familial ovarian cancer cases are mediated by high-penetrance mutations in the *BRCA1* and *BRCA2* genes. Only few other genes have been identified for familial ovarian cancer.

We conducted targeted next-generation sequencing of the protein coding region of 21 candidate genes, including UTR regions, in genomic DNA samples of 48 patients with familial ovarian cancer from the Republic of Bashkortostan. We identified deleterious variants in *BRCA1*, *BRCA2, CHEK2, MSH6* and *NBN* in a total of 16 patients (33%). The *NBN* truncating variant, p.W143X, had not previously been reported. Seven patients (15%) were carriers of the c.5266dupC variant in *BRCA1*, supporting a Russian origin of this founder allele. An additional 15 variants of uncertain clinical significance were observed. We conclude that our gene panel explains about one-third of familial ovarian cancer risk in the Republic of Bashkortostan.

**Supplementary Information:**

The online version contains supplementary material available at 10.1186/s13048-023-01119-z.

## Introduction

Ovarian cancer (OC) is the third most common gynecological malignancy following endometrial and cervix cancers. Annually more than 295,000 new cases of the disease and 184,000 associated deaths are registered worldwide [1]. In Russia were registered 14,318 new cases of ovarian cancer and 7,616 deaths in 2018 [2]. The high mortality in ovarian cancer rate can be attributed to the asymptomatic nature of the disease in earlier stages and lack of effective screening methods [3].

Ovarian cancer is polygenic in nature. Genetic factors have an important impact on OC etiology. About 5–10% of all ovarian cancer cases are familial, and about 15–25% of hereditary ovarian cancer (HOC) cases are mediated by high-penetrance mutations in the *BRCA1* and *BRCA2* genes [4, 5]. According to the ClinVar database, about 3,000 and 3,400 pathogenic sequence variants (PVs) and likely pathogenic variants (LPVs) are known in *BRCA1* and *BRCA2* (https://www.ncbi.nlm.nih.gov/clinvar). However, additional risk genes for ovarian cancer have been identified encoding proteins involved in homology-directed repair proteins such as PALB2 [6], BRIP1 [7], RAD51C [8], RAD51D [8], or in mismatch repair such as MSH2 or MSH6 [9]. Furthermore, a major fraction of the remaining OC risk is due to sequence changes at other genomic loci with susceptibility variants of moderate to low penetrance [10]. High rates of morbidity and mortality from this cancer type indicate the need for a deeper understanding of the disease molecular genetic basis, which in turn will contribute to the development of new approaches to the diagnosis and treatment of OC.

Attractive methods for searching gene variants involved in the cancer pathogenesis are next generation sequencing (NGS) technologies, which allow the simultaneous analysis of millions of DNA samples. One of the widely used NGS technologies is targeted sequencing. This approach allows the simultaneous analysis of several genes. Using targeted sequencing, some researchers have identified the mutational spectra of genes associated with breast and/or ovarian cancer and reported pathogenic abnormalities in genes (*CHEK2, ATM, NBN, RAD50, RAD51C, RAD51D, BRIP*, etc.) involved in cell response to DNA damage, homologous recombination repair, cell cycle checkpoint, or apoptosis with hereditary ovarian cancer [11–13]. Pathogenic variants in genes whose protein products are involved in Fanconi Anemia (FA) signaling pathway and the mismatch repair pathway (MMR) were also identified in patients with breast cancer and ovarian cancer. In recent years, several studies have been published in which patients with hereditary breast and ovarian cancer (HBOC) were investigated using targeted sequencing not only in the *BRCA1* and *BRCA2* genes, but also in other candidate genes. For instance, the Ovarian Cancer Association Consortium has sequenced several dozens of candidate genes in more than 3,000 unselected ovarian cancer cases and 3,000 healthy controls [6, 7, 14]. However, there are noticeable differences in the distribution of the spectrum and frequencies of genetic variants between different regions and populations in patients with ovarian cancer, which can be associated with the accumulation of genetic disorders in the population. In this research project, we included women with a diagnosis of hereditary ovarian cancer from the Republic of Bashkortostan to determine the mutational contribution of 21 candidate genes involved in carcinogenesis to the development of ovarian cancer in our population.

## Materials and methods

### Patient samples

All OC patients (*n* = 48) originated from the Volga-Ural region but belonged to different ethnic groups from Bashkortostan, including Russians, Tatars, Bashkirs, Ukrainians, and patients of other or mixed ancestry. The average age of disease manifestation was 44 years (19–74 years). The selection criteria of patients were the characteristic generally recognized signs of likely hereditary OC: burdened family history—cases of ovarian, breast, prostate and pancreas cancers in relatives of the first and second degree of kinship; primary multiple meta—or synchronous malignant neoplasms (polyneoplasia) in the patient herself; platinum-sensitive recurrence, young age of the patient—up to 45 years in conjunction with at least one of the above diagnostic criteria, platinum-sensitive relapse. Peripheral venous blood was taken by employees of the State Autonomous Institution of Health Republican Clinical Oncology Center of the Health Ministry of the Bashkortostan Republic (Ufa). All participants of this research signed voluntary informed consent for molecular genetic studies. This work was approved by the bioethical committee of the Institute of Biochemistry and Genetics, Ufa Federal Research Center of the Russian Academy of Sciences.

Patients had different histology type of tumors but 46 were epithelial ovarian carcinomas. Of these, 30 (65%) were serous tumors; 8 (17%) were mucinous tumors; 2 (4%) were mixed epithelial tumors; 2 (4%) were undifferentiated carcinoma; 1 (2%) was a clear cell tumor; 1 (2%) was an endometrioid tumor; 1 (2%) was a squamous tumor; 1 (2%) was a Brenner’s tumor. Stromal tumors were found in 2 (4%) cases: one granulosa cell tumor (2%) and one tumor from Sertoli-Leydig cells (2%). Tumors were predominantly of a high grade (G3-G4) – 36%. A low-grade tumor (G2) was detected in 32% cases, and grading of cancer cells was not histologically determined in 32% patients. Bilateral ovarian cancer was present in 2 (4%) women with OC. Stage I of disease was established in 16% of patients; II – in 11%; III – in 70% and stage IV – in 3% of cases. Seven patients (15%) also had a personal history of breast cancer, cervical cancer or colon cancer. Metastases were detected in 43% of the patients.

### Methods

Genomic DNA was isolated from peripheral white blood cells by routine phenol–chloroform extraction. To screen germline variants of the nucleotide sequence, the method of targeted next-generation sequencing was applied on the Illumina MiSeq platform using the AmpliSeq protocol with a custom panel containing primers for the synthesis of 661 amplicons covering the protein coding region of 21 candidate genes, including their UTR regions: *BRCA1*, *BRCA2*, *BARD1*, *BRIP1*, *CDH1*, *CHEK2*, *EPCAM*, *MLH1*, *MRE11A*, *MSH2*, *MSH6*, *MUTYH*, *NBN*, *PALB2*, *PMS2*, *PTEN*, *RAD50*, *RAD51C*, *STK11*, *TP53.* An additional file with used primers shows this in more detail (see Additional file 1). Then, an assessment of the reading quality and secondary processing of data was carried out in the multifunctional online service Base Space Illumina (https://basespace.illumina.com). An individual summary file was obtained for each sample under study, containing information on the number of reads, the percentage of Q30 bases, the percentage of gene coverage, the percentage of aligned reads, the level of autosomal variants colling, the number of identified SNVs, deletions, insertions relative to the reference genome, the gene regions are indicated in which changes were detected, etc. Also, for each sample,.vcf and.bam files were obtained containing complete information about all detected changes in the studied genes. For bioinformatics analysis of the nucleotide sequence variants, Illumina Variant Interpreter, ANNOVAR, SNPeff, ClinVar, gnomAD, ExAC, 1000 Genomes and ALFA services were used. Changes, detected by Targeted Next-Generation Sequencing of 21 candidate genes in hereditary ovarian cancer patients, were annotated in ANNOVAR program, using the summarize_annovar.pl script. It makes possible to compare single nucleotide substitutions with a number of specialized databases and predict the functional significance of the detected changes using in silico tools (SIFT, PolyPhen-2, LRT, Mutation Taster, Mutation Assessor, ClinVar, phyloP, GERP ++ and others) from dbNSFP v.1.3. In addition, the CADD (Combined Annotation Dependent Depletion) program was used. To estimate the population frequencies of the identified variants, we used data from the 1000 Genomes project, the Exome Aggregation Consortium and Allele Frequency Aggregator.

After ANNOVAR annotation, a search for pathogenic variants was conducted that may represent driver mutations in the development of ovarian cancer. This further analysis included the use of custom filters, based on the following criteria:

1. The selection of variants located in exons and splicing sites,
2. Selection of potentially functionally significant genetic variants: truncating variants (frameshift, stop gained and splice variants) and nonsynonymous single nucleotide substitutions,
3. Selection of variants with frequency no more than 1%, according to 1000 Genomes, the Exome Aggregation Consortium and Allele Frequency Aggregator. Previously undescribed variants with unknown frequency were not rejected if they had potential functional significance. Verification of all selected nucleotide sequence changes was carried out using Sanger sequencing. The frequencies of identified variants were calculated as the ratio of the samples number with the variant to the total number of samples.

## Results

**Fig. 1:**
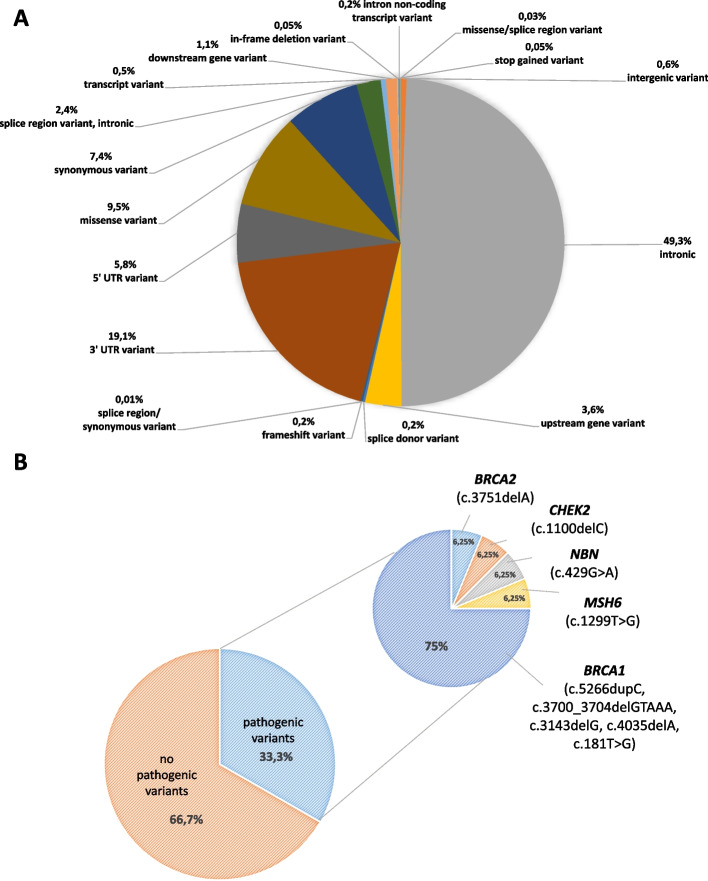
**A** Spectrum of variants detected by targeted next-generation sequencing in hereditary ovarian cancer cases. **B** Distribution of patients with and without pathogenic or likely pathogenic variants

By sequencing 21 candidate genes in 48 ovarian cancer patient samples, an average of 181 variants (range: 122–226) were detected per patient. Most of the variants (on average 88 variants per patient) were identified in the intronic region of the genes; variants of the 3’-UTR (on average 33 variants in patient) were also often found. Any variants of the 5’-UTR; missense; synonymous; upstream gene; splice region/intron variants also were detected in all patients. The distribution of identified variants among different portions of the respective genes is illustrated in Fig. 1A.

Pathogenic (PVs) and likely pathogenic variants (LPVs) of *BRCA1*, *BRCA2*, *CHEK2*, *MSH6* and *NBN* genes were detected in 16/48 patients (33%). The vast majority of PVs/LPVs were found in *BRCA1*, in 25% of the patients (12/48). In one patient we observed PVs/LPVs in *CHEK2*, in one case in *BRCA2*, in one patient in *NBN* and in one case in *MSH6*. No pathogenic or likely pathogenic variants were found in *BARD1*, *BRIP1*, *CDH1*, *EPCAM*, *MLH1*, *MRE11A*, *MSH2*, *NBN*, *PALB2*, *PMS2*, *PTEN*, *RAD50*, *RAD51C*, *STK11*, or *TP53*. The distribution of pathogenic or likely pathogenic variants in candidate genes is illustrated in Fig. 1B.

### Loss-of-function variants

Functional disease-causing variants (frameshift, stop gain and one deleterious missense variants) were found in 33% of patients. In total, 9 different loss-of-function variants were detected in 16 DNA samples (Table 1). By far the most frequently mutated gene was *BRCA1* with the founder mutation c.5266dupC in seven cases, the variant c.3143delG in two patients, two further PVs (c.4035delA, c.3700_3704delGTAAA) in one patient each, and the variant c.181T >G (encoding the RING finger substitution p.Cys61Gly) in one case. In the *BRCA2* gene we found one pathogenic variant (c.3751dupA). In the *CHEK2* gene we detected one patient with the founder mutation c.1100delC. In the *NBN* gene we detected a novel truncating variant (c.429G>A, p.W143X). One further truncating variant was identified in *MSH6* (c.1299T>G, p.Y433X) (Table 1).

**Table 1:** Loss-of-function variants in familial ovarian cancer patients from Bashkortostan

Gene	Variant	Exon	Protein change	Zygo-sity	Type of variant	ClinVar
*BRCA1*	c.181T>G	4/23	p.Cys61Gly	Het	missense	pathogenic
*BRCA1*	c.3143delG	10/23	p.Gly1048ValfsTer14	Het	frameshift	pathogenic
*BRCA1*	c.3143delG	10/23	p.Gly1048ValfsTer14	Het	frameshift	pathogenic
*BRCA1*	c.3700_3704 delGTAAA	10/23	p.Val1234GlnfsTer8	Het	frameshift	pathogenic
*BRCA1*	c.4035delA	10/23	p.Glu1346LysfsTer20	Het	frameshift	pathogenic
*BRCA1*	c.5266dupC	19/23	p.Gln1756ProfsTer74	Het	frameshift	pathogenic
*BRCA1*	c.5266dupC	19/23	p.Gln1756ProfsTer74	Het	frameshift	pathogenic
*BRCA1*	c.5266dupC	19/23	p.Gln1756ProfsTer74	Het	frameshift	pathogenic
*BRCA1*	c.5266dupC	19/23	p.Gln1756ProfsTer74	Het	frameshift	pathogenic
*BRCA1*	c.5266dupC	19/23	p.Gln1756ProfsTer74	Het	frameshift	pathogenic
*BRCA1*	c.5266dupC	19/23	p.Gln1756ProfsTer74	Het	frameshift	pathogenic
*BRCA1*	c.5266dupC	19/23	p.Gln1756ProfsTer74	Het	frameshift	pathogenic
*BRCA2*	c.3751dupA	11/27	p.Thr1251AsnfsTer14	Het	frameshift	pathogenic
*CHEK2*	c.1100delC	11/15	p.Thr367MetfsTer15	Het	frameshift	pathogenic
*MSH6*	c.1299T >G	4/10	p.Tyr433Ter	Het	stop gained	pathogenic
*NBN*	c.429G >A	4/16	p.Trp143Ter	Het	stop gained	pathogenic

The seven patients heterozygous for the c.5266dupC mutation were diagnosed with serous (5/7), clear cell (1/7) and squamous cell (1/7) carcinomas. Two patients with c.5266dupC additionally had cervical cancer and/or breast cancer, as well as vaginal, omental and liver metastases. Five c.5266dupC carriers were of Russian origin and two carriers of Tatar origin. One of the two patients with BRCA1*c.3143delG also had breast cancer. The patient with the MSH6*p.Y433X truncation also had endometrial cancer, this patient also harbored unclassified variants in *MUTYH* and *BRCA2*. The patient with CHEK2*c.1100delC was identified with serous carcinoma. The clinical data of these and the remaining PV carriers are summarized in Table 2.

**Table 2:** Clinical data of patients with identified loss-of-function variants

TruncatingVariant	Other variants identified	Histology	Subtype	Grade	Other cancers in the patient	Metastasis
*BRCA1* c.5266dupC	–	epithelial	serous	Gx	–	–
	–	epithelial	clear cell	G_2_	–	–
	–	epithelial	serous	G_2_	–	–
	–	epithelial	serous	G_2_	–	omental metastases
	*BRCA2* c.4043G>C	epithelial	squamous cell	G_3_	cervical cancer; breast cancer	liver metastases
	–	epithelial	serous	G_4_	breast cancer	–
	–	epithelial	serous	Gx	–	vaginal metastases
*BRCA1* c.3700_3704delGTAAA	*ATM* c.2149C>T	epithelial	serous	G_4_	–	omental and mesenteric metastases
*BRCA1* c.3143delG	*PALB2* c.1486G>C	epithelial	serous	G_2_	–	–
	–	epithelial	serous	G_2_	breast cancer	lymph node metastases
*BRCA1* c.4035delA	–	epithelial	serous	G_3_	–	omental metastases
*BRCA1* c.181 T > G	–	epithelial	mucinous	G_2_/G_3_	–	omental metastases
*BRCA2* c.3751dupA	–	epithelial	serous	G_2_	–	–
*CHEK2* c.1100delC	ATM c.6028A>G	epithelial	serous	Gx	–	–
*NBN* c.429G > A	–	epithelial	serous	Gx	–	–
*MSH6* c.1299 T > G	*BRCA2* c.5624A>C; *MUTYH* c.985G>A	epithelial	mixed	G_2_/G_3_	endometrial cancer	–

### Variants of unknown clinical significance

We additionally identified 15 rare variants of uncertain significance, including six novel missense variants, that were located in *BRCA2 (3), PALB2 (2), ATM (3)*, *NBN (2)*, *MRE11* (2), *MSH6* (1), and *MUTYH* (2) genes (Table 3). Six of these variants (BRCA2*p.Cys1348Ser, BRCA2*p.Lys1875Thr, PALB2*p.Asp496His, ATM*p.Arg717Trp, ATM*p.Arg2010Gly, MUTYH*c.985G>A) were found together with truncating variants in *BRCA1, CHEK2* or *MSH6*, respectively, making them less likely to constitute the driver of carcinogenesis. The MUTYH*c.1187G>A variant, encoding p.Gly396Asp (rs36053993), was considered potentially pathogenic in the biallelic state [15] but the patient here was heterozygous only. One patient had three variants of uncertain significance in *BRCA2*, *MRE11* and *NBN*, illustrating the challenge to identify a causal variant among rare missense substitutions of different DNA repair genes found in the same patient.

**Table 3:** Rare missense substitutions of different DNA repair genes

Gene	Variant	Location	Type of variant	Protein change	Pathogenic variants in other genes	Databases		
						**dbSNP**	**ClinVar**	**gnomAD MAF**
*ATM*	c.2149C > T	ex.14	Missense	p.(Arg717Trp)	*BRCA1* c.3700_3704delGTAAA	rs147515380	Uncertain significance​	0.00003
*ATM*	c.6022A > G	ex.41	Missense	p.(Ile2008Val)	–	rs2084586855	Uncertain significance​	–
*ATM*	c.6028A > G	ex.41	Missense	p.(Arg2010Gly)	*CHEK2*c.1100delC	–	Uncertain significance	–
*BRCA2*	c.3968A > G	ex.11	Missense	p.(Lys1323Arg)	–	–	–	–
*BRCA2*	c.4043G > C	ex.11	Missense	p.(Cys1348Ser)	*BRCA1* *c.*5266dupC	–	–	–
*BRCA2*	c.5624A > C	ex.11	Missense	p.(Lys1875Thr)	*MSH6* c.1299 T > G	rs587782583	Uncertain significance	0.00001
*MRE11*	c.1480G > A	ex.13	Missense	p.(Glu494Lys)	–	rs104895016	Conflicting interpretations of pathogenicity​	0.002
*MRE11*	c.1492G > A	ex. 13	Missense	p.(Asp498Asn)	–	rs564511708	Uncertain significance​	0.002
*MSH6*	c.926C > G	ex.4	Missense	p.(Ser309Cys)	–	rs544222338	Conflicting interpretations of pathogenicity	0.002
*MUTYH*	c.985G > A	ex.11	Missense	p.(Val329Met)	*MSH6* c.1299 T > G	rs147718169	Conflicting interpretations of pathogenicity	0.00008
*MUTYH*	c.1187G > A	ex.13	Missense, Splice region	p.(Gly396Asp)	–	rs36053993	Likely pathogenic	0.003
*NBN*	c.515T > C	ex.5	Missense	p.(Val172Ala)	–	–	–	–
*NBN*	c.1912T > C	ex. 12	Missense, Splice region	p.(Ser638Pro)	–	rs199657566	Uncertain significance​	0.00003
*PALB2*	c.315G > C	ex. 4	Missense	p.(Glu105Asp)	–	rs515726108	Uncertain significance​	0.00003
*PALB2*	c.1486G > C	ex.4	Missense	p.(Asp496His)	*BRCA1* c.3143delG	–	–	–

## Discussion

The present pilot study aimed to investigate the mutational spectrum of 21 candidate genes in 48 patients with likely hereditary ovarian cancer and to identify major contributing genes in the hitherto uncharacterized population of Bashkortostan. The results show that about one-third of these ovarian cancer cases from the Volga-Ural region can be explained by a truncating mutation in one of these genes, most notably *BRCA1*. Of note, the common truncating variant c.5266dupC accounted for about 1 in 7 ovarian cancer cases in our cohort, supporting its predominant role and proposed origin in Russia [16]. A previous breast cancer study from Bashkortostan identified c.5266dupC in some 4% of breast cancer patients [17], indicating a three– to fourfold enrichment of pathogenic *BRCA1* variants in ovarian cancer compared to breast cancer from the same population. This is consistent with previous comparative studies in Slavic breast and ovarian cancer patients, e.g. in Belarus [18]. Our high frequency of c.5266dupC is also consistent with a previous study of Suspitsin et al. who found this variant in 9.7% of ovarian cancer cases from the North-West and 17.2% ovarian cancer patients from the South of Russia [19]. The latter matches our findings and is clinically important because *BRCA1*-deficient ovarian carcinomas are particularly vulnerable against platinum-based therapy as well as PARP1 inhibitors [20–22]. Three of our *BRCA1* mutation carriers also had breast cancer (and one additional cervical cancer), and one *MSH6* mutation carrier also had endometrial cancer, in line with the known role of these genes in different types of DNA repair and cancer predisposition.

Apart from *BRCA1*, *BRCA2* and *MSH6*, no clearly pathogenic variant was identified in other established ovarian cancer genes tested. We identified one patient with a well-known truncating variant in *CHEK2*. Although *CHEK2* variants have been proposed to predispose to ovarian cancer [23] and the c.1100delC variant has been previously reported in two Russian ovarian cancer patients [24], there is insufficient evidence at present to conclude that *CHEK2* contributes to ovarian cancer risk as it does in breast cancer [24]. An additional missense variant of ATM in this patient was of uncertain clinical significance. We furthermore identified a novel truncating variant in *NBN,* another candidate gene for ovarian cancer. NBN encodes Nibrin, the Nijmegen Breakage Syndrome protein, which recognizes DNA double-strand breaks and modulates homologous recombinational repair [25, 26]. It is unclear whether *NBN* represents an ovarian cancer susceptibility gene [27–30], though a lack of the MRE11-RAD50-NBN complex has been reported in almost half of epithelial ovarian cancers [31]. However, such deficiency may occur by somatic inactivation and much larger case–control association studies will be needed to finally resolve the role of *NBN* germline variants in the etiology of this cancer.

Apart from the uncertain role of some of the candidate genes selected for panel testing, the interpretation of missense variants is another challenge that will need to be addressed in the future. Unclassified variants have been found in several patients here, including one patient with three such variants. Such rare variants could make a significant contribution to those two-thirds of hereditary ovarian cancer patients that are not explained by PVs in the currently tested genes. However, it is not possible at present to assign a risk estimate to any of these single variants nor to their potentially synergistic combination.

In summary, this is the first report of multi-gene panel testing for germline variants among cancer patients from Bashkortostan. This study has identified *BRCA1* as the main contributor to the familial ovarian cancer risk in this country and has uncovered novel variants in additional genes that will deserve consideration in further studies of hereditary ovarian cancer.

## Supplementary Information

**Additional file 1.** List of primers to cover protein-coding and UTR regions selected for targeted NGS sequencing for the AmpliSeq Illumina panel.

## Data Availability

Raw data of Targeted Next-Generation Sequencing available at the link https://www.ncbi.nlm.nih.gov/sra/PRJNA906939.

## Abbreviations

CADD: Combined Annotation Dependent Depletion
DNA: Deoxyribonucleic acid
FA: Fanconi Anemia
HBOC: Hereditary breast and ovarian cancer
HOC: Hereditary ovarian cancer
LPV: Likely pathogenic variant
MMR: Mismatch repair
NGS: Next-generation sequencing
OC: Ovarian cancer
PV: Pathogenic variant
SNV: Single-nucleotide variant
UTR: Untranslated region
