# Supplementary material for: Targeted next-generation sequencing of 21 candidate genes in hereditary ovarian cancer patients from the Republic of Bashkortostan
Source: J Ovarian Res. 2023 Apr 4;16:66. doi: 10.1186/s13048-023-01119-z (PMC10071671; doi:10.1186/s13048-023-01119-z)
Supplement: Supplementary file 1 — Additional file 1. List of primers to cover protein-coding and UTR regions selected for targeted NGS sequencing for the AmpliSeq Illumina panel. [file 13048_2023_1119_MOESM1_ESM.pdf]

Additional file 1. List of primers to cover protein-coding and UTR regions selected for targeted NGS sequencing for the AmpiSeq Illumina panel

Target ID	ULSO sequence	DLSO sequence
AMPL193028	GAGAGAGGACAAGGAGAGGATTCTC	TTCGAACTCAGGTGATTCTGGCCTC
AMPL193027	AAAGTACTGGGACATGAAGTTAAGG	ATGCCTGTACTGAGTTTTGTGGGAT
AMPL90450	TTGGCTTTTGAGGCTATATCCACAG	TCCTTGTGTTTCCTACATGTTCTAC
AMPL193029	GAGGCCCAACTCAGGTACCTGGATA	ACCTTCTCTCACATCAAGCTGACAT
AMPL193026	CGGCCAAAGCCCACTCTCTAGGTTG	AGAGAGAATGGAGGGAATCGGCAGC
AMPL193022	CCGGCCAGGAGATTTCAACCAAGCA	AGTAAGATTCTGCAGAACCCGGCCA
AMPL193030	ACAGCAGTGTTCCCTTCTTTTAGGG	CTATGACACTCAACCCTGTGCCTCT
AMPL193023	TCTTGGGTGTCTTATAATCTTGAGT	GGTAACAAGGGTGCTTCAGGGGTGT
AMPL193024	ACCCACAGGAGGTGAATCAACTCTG	ATCAGGTGGTAGAGGAGCTAGGGGG
AMPL193018	ATTGACAGGCAGAAGATGAGATGGA	TTCTGCCTGCCTGTGGCTATAGAAG
AMPL193016	TGCTAAGCTGGTACGACCAAGAGAA	TATACCGGATGGATGCAGGTGACTC
AMPL193017	ATCTTGAGAGTGCTTGAGAGAAGCT	GAGACTTACTAAGGGCCAGGTGCGG
AMPL742144	TGAGGGACACACTGTCTCTAAAAAC	GGACATTTCAGTCAAGACTTGAAAA
AMPL742143	CCGATGGGTCGAACTTCCGTTCAGA	ACTAGGAGAGGGTCGTGTGGGGGAA
AMPL742146	TTGAGGAGCCTCTAGAACTATGAGC	TAATTGCCTATTGGCCTGTGCTGCC
AMPL328824	CGTTATGAGCTCCAGACTACATCTC	AGAGGCTTTGAAGGCTACCTCTGGG
AMPL1140917	AAGGTGCTCGCTCGCCGAAGGACTA	CTCTGAAGCCTTGAAGAATGGGGCC
AMPL193682	GGTCCCCTCCCTATTATGCCCGTTT	AAGGACTAGCGCTCTGTGCGCGCGG
AMPL193679	TCCCACACTTACTTTGTAACATGAT	ACAAAACCTTAAAAAGTGGTTCTGA
AMPL193675	GTTTTGAGCCATTCATTTCTGCCTT	ATTCACAAAGCAGTTTACGGCCAGC
AMPL193676	CTCATATCTTCTGAATTCTAAACCA	ATTTGGCAGCCACTGAAAAAGAAAC
AMPL193677	GTGCTATTCTTGCAACTCTATTTCA	TCTTCCCTTACACATCCTTCATAAA
AMPL193683	CACATTAAGTACCCAGATCCGAAAC	TGATTCAGGCTGGAATCATCCATTG
AMPL193678	GTGACAGGGAGGACAAAATTAACGG	TCTACATGCATCACTGTTCCTTTCC
AMPL193680	CGCCCAGCCACTATTACTTTTTCTT	CAAAAGAACCGCTGCCAAGAATCTT
AMPL193681	AGAGTACATTCTAAGACTGTCCCTA	AAAATTTCACACCAGTGGAGTGGCT
AMPL742124	TCATGACCTTCAAAGATGTCTTCGT	CAAAGTGACTAGGGCAAGTTGGAGG
AMPL742125	TACAAGACTCAAGTAAATAGAAAGG	ACATTTGTAATTTGTGTCCATTTGC
AMPL193063	TTCTCCGGCATGCCCTGAAAGAAGC	CCTTAGTAGAGCTCCTTTCTGTGTT
AMPL193051	CGGAATCCGCACAAGCACCAACGTT	ATGTCGAAACCTCCTCACCTCCTGG
AMPL193054	TCAAACCATTCTACTATCACAATCT	TATTAGCTGGACTTCATGCTGCTGA
AMPL193057	GCCTTTCCTAGGCCTGGAATCTCCT	GCCAGGAGAAGCCTAAGTAAAAATA
AMPL193061	TAAATGTCTTTTGTGGAAAAGTCAG	TGCAAGACACTGATATGATGTACTG
AMPL193062	TATCATTGATACACAGTTTAGGTTT	TCTGTGATCAGAATTCCTCCTCTTT
AMPL193045	GAAAAATACAGCCATTTAAAGCTAG	GATTTCTATACCACTGGATCCAGTT
AMPL193064	TGGTATAATCATGTGGGTAACTGCA	TCCTAAGACCAACTTTCAACCTCTT
AMPL89312	CGACGAAGTAAATCTTCTTGTAAAG	AAAAAGCTTGAATCTGCTGGGCGCA
AMPL89313	CTTTATGAGGACAGCACATTGCCAA	TCTGCATCTTCTACAAAAGCTTCCA
AMPL193050	CAAAGGTGCTACAATTAGATACTAA	AGCATCATTTGATCCAAAAGGTACA
AMPL193046	ATCTTCTTTTCCAAGTCATTCATTA	GAAACAGTTACAGTGCTGATGATGT
AMPL193047	GGGAACTTATAAAATAATTCTGGCA	TCACTGAGATTAGGATCAAATGAAG
AMPL193048	GAAAGTAATATCCAAACTGTGCACT	TTCTGGGTAAACCCATCCAAGGCTG
AMPL193049	ATCATGTTAGAGCATTTAGGGAATT	TAATCTGTTTGCCAGGGTCCAAGCC
AMPL193053	ATGGCTTGCGAAAATAACAAAACAC	ACAACTCCTTAGGGACAAATGTAGT
AMPL193058	CAACAGCATCTAGCTGAGCTAACAC	AAACAGAGTACTGGCTGGGTTGGGT
AMPL193059	AGAACTGGGAATTTTCTCCATCAAT	TCTGCATTGGTTCTACATAGCCTGT
AMPL193055	AAATATACTTTTCCTTCTCACAGGA	GGATTGTTAGTCCTAAACTGTACAC
AMPL193060	AAGTTTCCCATTACCAAGTTCTGAA	TCCCATCACATAAAATGTGGTAACA
AMPL193052	CACTAGAAACACAGAGGAAAACAAA	TGTAAATGGAATTTATCCTTCTCAC
AMPL573938	CTATTACTTCAGCTTTTAGCTGTTT	TGCACTGTCTAGTTCCCAAATTCCT
AMPL573939	ACATCACTTATTATTGCCTATGTCA	TGATGTTTTCTTCTGACATTTCAGT
AMPL323324	CTGAGCTATTTGTTTCTTCCAAAAT	TCCTCAGTTACAGCTCTCATTTTTC
AMPL1140911	GCCCCAGTGGCCAATCAACAGGCGC	CGAAAGGCCTCGGAAAGGGGCTCGC
AMPL1140912	GCCCTCCGTTGAGGTTCTTCGCCTT	AAAGCACCGCATCTACCGCGCGGCT
AMPL91660	CCCTGCACTCATTCAAGCCAACTCT	CTTGTTGGCATCACTCAGCGCCGGA
AMPL573932	TACCTCTAAGTGTCTACCTCGATCG	TCTCCTCCGACCCTGTTTACAAGCT
AMPL573933	TGCAGCCTACCTACCGAAGGACCCA	CCCAACTCACTATCTCCGCGCCGGC
AMPL91653	AAAAAAGTCTGCCTGTCTGTCTGTT	CAAATAAACAATTCCAAGTCTTCTG
AMPL91662	ATATATAAAACAAGGTATCTTGTTA	CCTTTGATTTTGAACCTAGAAAGAA
AMPL91673	GAATCTGATATGACCCTTCGTTTTT	CTAACATTTAAGTGTTCTGAGCACG
AMPL91674	AGGGCGTTTCCTTCCTAGAGCTTTT	CTACTTCGCCTAGATCCTTGTGTCT
AMPL91664	TGACAAGATCAAAGTTCTGAGACTT	AGCCATTTCCAGTCACCATTCTCTT
AMPL91665	ATCACCTTCCAGCACACTGTAAGTC	ACTTCCTCATCCCAGGAGTACAAGA
AMPL91666	CAAAGTTTTGGATGCATCCCAAAAC	GGTAATGATCCTACAGATCTCCCTC

AMPL90995	AGCTCCTGATCAATAAGGCATTTTT	TATCAGACCTTCCTGAAGAGAACAG
AMPL91667	GGCACAACCATGAGGTCTTCTATGG	ACACAACCACCTAGAGCAGAGAGGG
AMPL91668	AATCAGGAAAACGACCTTCAGGATT	TCATTAATAGCATAATGGTTACAGA
AMPL91669	CAGCTGGTAACGGTTCCTACCAATC	GAGAGATGACCTGCTTAAGGATTTT
AMPL91670	GCAAACAGCACTACTTATCAAAGCC	TATGGTCCTACAGCCAATTCTGTTG
AMPL91671	AGAATTACTGGGCGACACATAGGAC	CTAAAAGATAGAGCAGAGGCCAGGC
AMPL91672	TTGGAAAATGATCACCTAAGTATGT	CGACTATAGTTAGCCAGGCACAGTA
AMPL91661	AGTGATGTTTTTAAAGTAACACCAG	GCACTCTAAAACAACTTTCACAATC
AMPL91655	TCATCCACAAGCACCAGAGAATGTG	GTTAAGAGACCAGCCTAACATGGTG
AMPL91656	TGCTCTAAAAACATTCATATTGTTT	ATGAGTATGCTGGCAGTTTCACTTA
AMPL91657	TTTCTACCATGCATGCCTTAAGAAA	AGCAACATCGGTTAGTACATCCACG
AMPL91654	TTACTGTTTCTTTGAAACTTAAGGT	ACATACCATATGTCCTAGGCGCACA
AMPL91658	TGCATGTACCATAACATCTAGACTT	TTCATAGTGCATCATCCCTTCCCCT
AMPL1420985	AATAAGACGATTCACAGGTGAGAGT	AGTCAACTAATAGTCTTATGCCTGG
AMPL1420986	AAAATAGAAACCAGATGGGAAGTAT	TTTCCATGGGTCAGTGTGGAAAATG
AMPL1420987	TTCTCATGGCTATTTTCTAGTTCTA	GATTGAAATAAGGGTAAATGAGCAA
AMPL1420988	AAAATTGGCTTATTCTGTGCTGTTG	AACTGTTTCCCTTTTTCCTTGACAG
AMPL1420989	AGCAGTTTCATCTTTGCTTTTGTTA	ACATGACTTGGTGAGGAAGTCTATT
AMPL1420990	CACATTTCATTCTGATAGAAAATGA	GCAGTTAGTTTGTGTTAAGCTCTTG
AMPL1420991	AGACACTGAGAGACTTGCCAGTTTG	CCAAACAAAAATCCGAGGCGTTAGA
AMPL1420992	GAAGTTGGTGATTACTTGATTTACA	ACAAAAGAGGACAGTTTGATAACTA
AMPL324361	CCAGTCTAGCAAATTGTCAGTCCAA	CCAGATCATACAAAAGGCCATACAG
AMPL1420993	TTTCTACACTTTCATCCCTGTTTTT	AATTAAGAAGCAGTCTTCTTCTGGT
AMPL1420994	ATCTTGGTTAAATAGACAAGTGGTA	TTTTTGTGTGTGTGGTAAAAATCAA
AMPL1420995	TAATGCCAGATTAGGAATTCATGTT	TGAGTCTTTTAATATTCTCCATACC
AMPL1420996	CTGCACACAGTATATCATCTATGAA	TTAGAAGCTGGGATTGCTTTTAAAG
AMPL193584	TTCAGGTAAGGGATATTCAACTTGT	TTATCACCCAGAGAGGGTTCGGCAG
AMPL193588	AAACTGCCATGAAGAAGAAAAACCA	CTGAAAGATGATCACGATGAGCAGT
AMPL193585	TAAGTGTAAAAATTGTTGCCTTTGA	ATTTTGGAAAAGCTTCTGGCTGGGC
AMPL193575	TGGGTGTAGATTCAATGGAAATAAT	AGATGATCCTTTTGGAGAACTACCT
AMPL193580	TTGATTCTAGATTCTTCTGCCTAAT	GCTTACATTCACCAAACAATTATTG
AMPL193589	ACAATAAATTTACTGCCTGACGTTT	TCAAGGTGTGTGATAAAGCAGACTT
AMPL193574	CAGTAGCCTTAAGAGAAAACGATTT	TCTGCCACATATACTCTTTTTAGTC
AMPL193576	CCAGGTACACCACCTTCTACATTAA	CTACTTCAGTTGGAGCAGTGGCCCC
AMPL193577	GTCTCCAGACACTAAGAGCAGGAAT	TACAGGCGAGTGATGTCTAGTCCCT
AMPL193578	CCAAGTCCTCCTGCAGATGTTTCTG	TCCTGAGAAGGTCTGCAAAAATTAT
AMPL193579	GGGAGCAATAAATTTCATGTAACAG	AAGGCTTCTGCAAGATCTGGAAAAA
AMPL193586	TATGTTAGAATAACCTCTGCTCCAT	TCTGGAGTTCTGGAATACGTATTTC
AMPL193587	CCTTCTGCTTTGAGAAAGTGAGAAA	CAAATAAGAAAACCAAAGCTCAAAG
AMPL193581	AAGGCGGGACGATGCCGGATAATCG	TCACTTTATTTTTGGGCGACTTACC
AMPL193582	GCGAGTCCCTATTTTGGGAGCATTG	GGTAAAGACGGAGCTTCTTGGGGGT
AMPL573936	GCGGCAGCTATTGATTGGACAGCTT	AATGTGGATGACGCCCAAAAGAAGC
AMPL573937	GCCCGTTAAGTCGTAGCCCTTAAGT	AAATTTCTCAACTCTGTGGGTTGCT
AMPL573930	ATCTTTTAACTTCGCATGTTCTGCA	CTTTGATAGCATTAGCTGGCCGCTG
AMPL92077	AACAGTGCCCAGCAAATAATAGGTA	ACAAACAATAGTGCCTCATTAACGT
AMPL573931	TTTCCACACAGAAAGACAAAGGATT	TGAAGCCTTCCTGAACTACTCAATT
AMPL92079	TCAGATTTGCATACATTTAACAAGT	TCCCATTTTCTTGATTTAATCCAGG
AMPL92060	AAAGATAACACTGGTGTTGAGACAG	TGCTTTGATGACATGTTGAAAAAGA
AMPL92065	CTTAGAGGATATCTTGGGACCTCCA	TCATTTTCCTGTTTATTTCTCCACT
AMPL92059	TGGCTAAACCTTGACCAGAAACTAT	ACAGTTCTAAGCACTGACTGGGGCC
AMPL92074	AGGATTTATTGTCTCATGGCTGAGA	GAAAAATAAACCAGCAGAAACTTCA
AMPL92073	ACTAAAGCAAACTCTTAACACACAT	ATCTCCACCAGCAAACTATTAAAAA
AMPL92058	CATGAGGTTTCTCCATGTTTAATCA	AAACTAACTTTGTGAAATGAGGGCC
AMPL92069	TCCTTTTGCCAGTGGTGTATGGGAT	GGAAGAAAGGTGCACCTTTACCTGT
AMPL92072	TAATACTTGCAAAGAAAGTTCCTAA	AGAAGATTTGAGGCCAGATGACACA
AMPL92061	CGGGAATCTGTACGAACCATCTGGT	TTCTGCCCGAAATTTGAAGTATAAA
AMPL92062	AGTTTTATTACAGAATAAAGGAGGT	TTATCACTACTTCCAGAAGTAGAAG
AMPL92076	GAGCTTGCCCAGCAAAACTGTAGTG	AACTTCTCTGTGCTGGTTCTACAGA
AMPL92067	GATTTGAAACCACATGTGTCTGACT	GCAAAGAAGCCTGAATTGAGCCTCC
AMPL92078	CCATATGCAAATCATACAATACAGC	CTGGTTAACAGTCTAACAAAATCTC
AMPL92075	CTGGAAATTTTATTTGAAGAATACA	AAGGAAGTGCCTGAACAATCCACAA
AMPL92068	CAGGCCTTCAGCTATCCCACCCTTA	TGACCACGTTACTTGATTGGGCTGG
AMPL92063	ACCTTGTTGGGTACAAAGAAATCCC	AAATGGGACTGCTGAATATTGCTGG
AMPL573927	ATATTTCCATCTTCTGTGAAATGTT	ATCTTGTGGGCTAGCCTCTCAGGGG
AMPL573928	TTTGGCATCTGAACTGACACAATAT	ATGTGTGAGCGCAAGGCTTTATAGA
AMPL1420968	GCCCTGCGGATCACGTGGGCCTCTA	CTCATTCTCTCTCTAACGTTCGTGT
AMPL1420969	AGGCTTAACTGAGGCCGAAGCAAGG	CGGGAAATCAGAGTCCCGCGGTGCG

AMPL1420970	TCCGAAAGATGTAGCGACCTGTAAA	AATCTCAGGGTCAGGAGGATCCGGG
AMPL253605	GACTTTCATTTCTCTTTATAGTTTT	GTGTTAATGCTGTAAGTTCACAGAA
AMPL253607	AAAACAACCATCAACTTACAGACCT	AGGGAACAAAAAGAAAAACATCCAA
AMPL253592	AAGCATCCAAATTGCAAACACAGTT	AAAAAGGCCCTTCACCTATCCCGTT
AMPL253591	AACTGGGCTTCCTTACTTGTAATCT	AGAAAGACAAGTGAAAGAAAATATG
AMPL116071	TCCACGATGTATACTTATATCAAAA	TCACAAGCTTTTTCCTTATATTGCT
AMPL253590	TTTTCCAATGTGGATGGCAAAATGG	TAGAGCAGGCCTAGAGAAACTGAAG
AMPL253611	CTCTGGTGATTGTGATATAAGTCAT	TTCGGCTATCCAAGGCTTTAATTTC
AMPL116146	TAAGTCCTTGGAGATTCTTCATTCA	TCAGTCCCTTGAAAAACCTGTGTAA
AMPL253610	TGGAGTCCAACCAAAAATTAAAACA	GATAGCTGCAGATTCACGAGTTATA
AMPL253603	CCAGTTTTCTTATCTCTTATCTCAT	ATTACTGTACTGAATACTTTGGCAA
AMPL253604	GAGTCAGAAGATCAAGATATTCAGA	TGTTTCAGAGTCTCTTTTTCTGCAA
AMPL253598	ATTTTTAAGGTTTCTACATTGCTGT	ACAGATTAGTGAGTCCTCAGTCAAC
AMPL253599	GGCTATTTTAAGTACCAGACAGTAT	TTGCTTAACTCACGTTCCTAGTCAA
AMPL116152	GGTTAATTCATCACTGTGCCTAGAT	AGAGTGGAGTACCAAGTCGAACAAT
AMPL253613	TGAAGTCTCCTTGTATGTAAGAACT	GATTTGTTCATCTTTGTCAGCCTAT
AMPL253595	TCCATAACCAGGTTTTTAAATTCCC	AAAAACAAGACAAATTCTGCCAACA
AMPL253608	CAAATCACTGATGACTTCTTGTAAC	CTTCCAAAGAGCATGATACATTCAG
AMPL253609	CTTTTACAATGTGCTGCTACATGTA	CTGAAAAACTCTCTGACAAACGGGG
AMPL253601	ACTCTCACACTTCAAATTCAAGCCA	AAGTGACATTGTGTTCTTTTCAGGG
AMPL253585	CATGTGCTCGCAATGTCAAAGTCTC	GCAATTCAAATGATGTAAACAAAAG
AMPL253586	GAACTTCAGTGGATAATTCCACAGT	CTTGATGTTCCCTGCTGGCCACTCA
AMPL253587	AACCAGCTTGTCCAATGGCGAAAAC	TTGCTGACGACGTTGCAAATTAGTG
AMPL253589	TGTCCGACGTGGTGCTATGAACATA	AGGACAGTACCATCATTTTCAGTCC
AMPL253584	TTTCTTTGTGTTTCTCGCATTCACT	CTGAATTTTATGATCGCAGACAAGT
AMPL253588	TTCCTTTTTGCATTTCTATTCAATG	TTCTTATAGTCGTCTTTCCCATCTT
AMPL253600	CAAAGGATACCAGGCTGAGGCTAAA	TCAACAGAGGGAGAATAAGCCAGTG
AMPL253597	CCAGTCTAATACTTACTGGTCAAGA	GGTTAAGTATCATTCTCCTTAGCAA
AMPL253612	CCAGGGAGGTAATGCTGGCATGATG	CAAAACTTTGCTGCTTGGCTAATAC
AMPL253606	AGAGCTTCCCACCATTCAGGTGTAG	ACAGATGAGGCTGACATTACCAGAG
AMPL253602	GTGGGGAACACAGCTAGAGAACGTT	AACGTCACAGGTTCACTGTTAGGTA
AMPL1420959	GACCTACATTTCTATGGCATTTAAA	ATGAACTTGTGCTGTGTGTCAGGGC
AMPL1420960	AGGCAGTAATTATCTCCTTCTCAAC	TTTAATGAACATTGAATCCCAGGGA
AMPL1420961	GAGAGCTCTGAGTCTGGATGACCAT	AAGAAGCATGCATTTGCACTGGGGA
AMPL1420962	TCAGCTGGCAATTCCAGGAAAAACT	ATGTAGGAGAAGAAGGTGGTGGGTA
AMPL1420963	CCTTTGCTGTATAAAGTAACAGTCA	GCTTTTCTTAGAAGTGGCATCTGAA
AMPL1420964	CTAAAGCTAGGAGATGTTGAATTTT	AATCAGGACCTGTCTATCTTTGGGG
AMPL1420965	TTGTTATTCTTTGCTGCAACAACTA	TCATTTTCTGAAATCTTGCATTCTT
AMPL1420966	TGTGATGGCTATTCATTCCAATGTG	AACATGTTGATAGAAGGTAACTGGC
AMPL1420967	ACAAATAAGAGGGAGTGACCAAGCT	TATTTGGAGACTGATCTAGAAGCAG
AMPL573941	CATCACACCCATTTAAAAGTGATCT	TAAAAATAAGAACACTTGGGCTGGG
AMPL573942	AGAGCTGCCATTCTGACGTCAAAAA	TCAAACCAGATGGAGCATTGCTTGC
AMPL92312	GCCCGGCCTCAGATGTTCATCCTAA	TAAACGCCTTTGTGATTGCCAGGGG
AMPL91384	CTAAATGTTTGACTTCAGGATATGG	GAACTATAGCGGCTGGGTGTGGTGG
AMPL92327	CTTTATTTTGTTTTTGTATTGTTTG	GATCTAATAGAGGCCAAGGCAGGAG
AMPL92317	CCAGGAAGATACCGGATGTAAATTT	CCGAAGATGAACTAAGAAAAGAGAT
AMPL92318	AAGGGGTATGCTGTCTTCTAGCACT	TCAGCCAACTAATCTCGCAACCCCA
AMPL92319	TGTTCATCTGTGTAATTAGCATGTG	TCAGGTGCCATCTCTGACAAAGGCG
AMPL92314	GAGACGCTGTCTGAAAATAATAATT	GCTTCTAAAGCTTTTAGCTAATGTG
AMPL92313	CATGCTGGTTGGTTAGAATAGAATG	GCTCTGCTATGACTGGAATGAGATA
AMPL92315	CTTTAAGAATGTGCCATGTGATCGT	AGCTTCAGATGTTTCAACTGAAGAC
AMPL92316	GTAATGTCAATAGCTTGACTAAGAA	GTGATTCTAAGGCTAATAGTTTCGT
AMPL92321	CTTGCGTTGGATGTTGTAACTTGAG	TTGTCCCTTCCAGTAGAATGGAGAA
AMPL92325	TGATCACAATGGGAAAATTATCCAG	GTGTTTATTAGGACTGGGCAACACA
AMPL92326	CTCCTCAACCATTTAGATCTTGATT	CAGCAGTTATTTTCCACACTACCTG
AMPL90475	GTTATAGCACTTGAGATATCTTAGT	GTTTTCTATCTCAACATCTCAACTT
AMPL92329	TTAAGTAAGTTGACTTTTTCTAATC	TACAATATTTTTATCCACTCACTTG
AMPL92322	AGTCTAAGCACTGCGGTAAAGGAGT	ATATTTAATTTGCAGAACAGCTGGG
AMPL92323	ATGTTTTCTCCATGTGGATCTTAGT	AAGCACTGCGGTAAAGGAGTTAGTA
AMPL573943	TCTTTGACGTCACGAAGTCGACCTT	TCAGTGTTGCAGCGATCTCGTGCCC
AMPL496117	TATCCAGTCATTGTAACACTGTTTA	TGACACAGTAGCCTATAGTTGGGCA
AMPL496118	AGATGCAAGTCTCTTGTCGGATATA	ATCCTTTCCTCATTAAATTACCTTG
AMPL496119	ACATATGGTCTCCATTTTACAGTTA	ATTACACCTAGTCTGTGGCTTGACT
AMPL496120	AAAGCTTCTCAGCCTTCCTAGGGAA	GGACAGATTAATATTGTTCTCTGTC
AMPL496121	AGTGAGGAATGGAGGCCATATTTCC	TGAGCCAATCTGCAATTTCTACTAC
AMPL496122	TTAGTTTTCACCAGAATGGAAAGAC	TGTAAACAGAAGCAACAGAAGGGAC
AMPL496123	ATTTTTGAGTCCTATTCAAATTCCT	TTTGGTGGTCTTACTGAGCTGGGTG

AMPL496124	TTAAGTTACAATGTTTTATGGCCTA	ACACAAATGATAGCATAACATATGC
AMPL496125	CTACAGTTTGAAAGTTCTTTACCTT	ATGTAACAATTGTTTGTTCTGTTTT
AMPL193509	GTGGTGACCTCCAGGATGTGGAAAT	TTGGCTATAGCATCGAGGTTTGGGG
AMPL193508	GGCACTTATGCATGATTTACCATCT	TCCTGAGTTACAGAGCATTAATCTA
AMPL193516	TTCCCAAATGACAAGTGACTTTTCT	CAGTGATGCTAAACAAAGATAAAAG
AMPL193512	CAAATGCCTGGTCATACATAACATG	TGCCTTGAAATGGATTGATCATGGT
AMPL193518	AATGGATGATGTGGCCATAGAAGAT	AGCATCCATTAATGTCTTTAACACT
AMPL193519	AGCTGTGTTCATTTTCTTGTTTTGT	ACAGTTATTCAAGGACACAAAACCA
AMPL193514	CCTGCAAAACAAGCTCTAATAATAA	TAGAGCCACTAGACAGTTTCTCCTT
AMPL193515	TGCTTTCTTGGGATGGTAAAATATA	CAAATACTTTGGCTAAGATGAGAAT
AMPL193525	ATGTGCAGTATTCCCTAGTTAATCC	AGAAAGCATGGAAGAATGGGATACC
AMPL193510	AAATTGGATTGGCGGTGATTTTCAT	AGTTCAGATCAAGCCAAGTATAAAG
AMPL90577	GCATTTGAAATTCTGTCTATACCAT	ATTACTGTGATCCTCAGGGCCATCC
AMPL193520	CTTCTATGTTTCCCAAATCAAATTC	TTTGTTGCTTTTCACCTAACAATGT
AMPL193521	CAGATAGTCACTCCGTTTACAATTT	TGATCATGGCTGTGATTACGGGGTT
AMPL193511	ATGGATGTAAACAGCCTCTTTGTAG	GGATGTAATAGTTGCTGTGTTATAG
AMPL193522	ACTAAAAATTGCCATCTCTGCAACT	TACCCACTCCATCCCTTTGTATACC
AMPL193524	TGGGAGGTTAGCCACCTGCCTATTG	ACAGTATAATTTAGTTCCTGCTTAG
AMPL1338740	CCTTGGGTGATAAAAGTGCTAAAAA	ACAGACTGCACCAAAGCAAAAAATT
AMPL193507	CACTTACATGTATGTGTGTGTTCGT	ACAGGATTTGTCATTTTATAGAGCT
AMPL1338741	CACATTTCAATACAGATTGTGCTGT	CTATTTTCCCGGTAGCGACTGCCTC
AMPL273654	CGGCTCAACTCTCAAACTTCCATCA	CTACTGAGCATGCCCAGTGTAGCTG
AMPL273655	GCCGAGGAAGAGGCTGCACGGTTAG	TCCGAGAGGAGAGAACTGAGCGCAG
AMPL273656	GCGGCGCACGGGAGGTTTAAAACCG	ACGAAGAGGAGGCGAGAAACGCCGC
AMPL193088	GGGAGAAGACGAATAATCCTCCGAA	CTTTCAGGAGAAGCCGAGGAAGAGG
AMPL193090	TCTAAGAGAGTGACAGAAAGGTAAA	TCTTCTGCAGGATGGAAATGGCTCT
AMPL193091	TCCATTAGGTACGGTAAGCCAAAAA	CACTATAGCTGGAGTAATAAAAGGT
AMPL193086	TTGAATCCAAAAACCTTAAAACAAA	AGCATTTAGTGCAAGTATACCTAGC
AMPL96430	TCTAACAAGCAGATAACTTTCACTT	AAAAGCCACCATTAACAAAAATGAG
AMPL90806	ATCTCACTCGATAATCTGGATGACT	AACATTGCCTGAATCTTTATAATGT
AMPL193079	ATCATTACACCAGTTCGTCCCTTTC	AGGTATTTAAAAGAAACACTGGATT
AMPL193080	AATTCTCAGATCCAGGAAGAGGAAA	TGCAACTGTGGTAAAAAGATAACCT
AMPL277289	ATATTACACAGCTACACAAAAACAT	GCACATGCTAAAATAATAGTACTGT
AMPL193081	CTGCATAAATTTCAAATGTGGTAAA	AACTGGGTCGTAGCCATTTAAGAAC
AMPL193085	CACCTGCAGATCTAATAGAAAACAA	CCTTTAACTGTCAAACTGTCAAAAA
AMPL193084	TATTCCTTGTCATTATCTGCACGCT	CATTCACATGATTTTTCTTCACTCA
AMPL90810	ATTAAACACACATCACATACATACA	ATGCTATCGATTTCTTGATCACATA
AMPL193082	GAAGTACAGCTTCACCTAGAGAAAG	AAGATGTTCAACATTTTCTTTCTTG
AMPL273627	ACTGGTAATCTGACACAATGTCCTA	TGAAAACCCACAAATATGACTCATC
AMPL273628	ATCAGTCTGTCTCCACTTTTTATAA	ATTCAAGTTTATTTTCATGGTGTTT
AMPL92667	TACATGCATTAGAATGTAGCAAAAC	AACGGGAAAGTGCCATCTTTATTAA
AMPL273629	TTTTCTAGCACCAATATGCTTTAAA	CTCTCTTAGCCAACTGCAAACTTAT
AMPL273630	GTTGTTGACTGATGTAGGTACTAAC	AAACAACAAGCAGTGACAGCGGCCA
AMPL273631	TGTGCAAAACTGCAAAATTCATTGT	ATTTTAGCACTGGCCTTGATTACAC
AMPL92673	TGTAGGACAATTTCTACTGAAAAAT	TAAGGTCAAAAGGGTGGAACGGCTG
AMPL273632	AAGGTATAATTTGAAGACACCAAAT	TTTTTGTGTGCAGCATTGAAAGTTT
AMPL273633	TAGTAGGTCCTATTCAATCTGTATT	ATTAGCTTTACAATAGTAGTTGTAC
AMPL273634	GCTTTTATGAAAGACAAGGTGGCAT	TAGGTCAGACCACAGCTAGTGAACA
AMPL273635	TCATTCAGACCTTCACATTCCAAAG	ATGGTGAGGTGTTAGAATAGTTGTC
AMPL273636	ACATCCAAAAAGTCTAAGAAAATAT	ACAGTGCAAAAGCCCATTATAATGT
AMPL273637	ACAGCCATCGTTATGAACATATTGC	AGAGATTACCTACAGAATGCATTTC
AMPL273638	CAGTGCATAGCATTTACACACAGAG	TTTTTAAAGCTGAAACATTTGATGC
AMPL273639	CATTAATTAAAACTTCAATGGACTT	TTGAACTTTTGAACTGGCAGGTAGA
AMPL273640	TACTCTACTCTCAGGCCATGTTTTT	ACTCTCATTTAATAAAACACCCACA
AMPL273641	GGCACAAGTCATGATGAGTAAATGA	TTTTTAAACAGTAGGCTTTGAAGGA
AMPL273642	AGATACCGAAAAATAAAATGTGTGT	ATGCTGATGGCAGAGTACAGAATAA
AMPL273643	TATTACAACGTACAAAGGACATGAT	TGAAAGGATATGCTTTAGTCTTCAA
AMPL273644	GGTACTGTCCTTACAACAGTTTGGA	AGCACATTTCTATTTTTCAAACATC
AMPL273645	CAACCAGAGTACTACCACCAAAGCA	TGTTTCCATTCACAAACATTTCTTT
AMPL273646	TGAATTCGAAAGGATTCTCAATAAC	TTGTCCTTTCCTGTCTTCACATTAC
AMPL273647	CTGATAAGCCAACAGCAGCCTTTTT	TGCATCTGTGGCATTAGAAAGTAAT
AMPL273648	TGCATGTTTTTATAAGCCCACTATA	TAATTTGCTCTAAATTGAGCAACTG
AMPL273649	CTTCATTCCACACAAATAAAATCTC	CGGTAATTTTCAGAGCTAAAGGTGA
AMPL277286	CAGAAAACCACACTGAAACATTTGA	AGCACAGATGAGAATCCTCATGCAA
AMPL277287	GTCATCCAGGATTAACATTTTCATT	GCAGTAAACAGACTTGGCAAACTGA
AMPL277288	TATATACAGCAGGGACTTGGTATTT	TGAAATTCCAATGAAAAATGTTTGC
AMPL1420972	CACAAGGTGGTGCTTCTGCCATTAG	TATCTCAATGGGCTGGGTGCAGTGG

AMPL1420973	TTCCTCGAAGTTCTAGGAAATTCTT	AGGATGCAACTGCAAATCTAAAATT
AMPL324332	TTGGTATAGGAATGTAGAAGTCTTT	TGGTGCTTAGAAAATTCAAGCATTT
AMPL1420974	AAAATTGCTCTTGGCTGTGAAAAGA	AAAGCAGCTGCATTTGCTCTTGGAG
AMPL1420975	ACATCTTTATTGAACTTGAAATCTA	CGATTCAGTTCACCGCTAAGGAAAG
AMPL1420976	GGCCCGACATTTCTTCATAGGTAGA	ATTTGAACAAAGCAGAAGGTTAAGT
AMPL1420977	TGCCAGGATTTCTCATTAACAATAT	TGACTCAGTGATACTCATGACCATT
AMPL1420978	TCCCATTAAGTGGTAGTAATTTCCT	TTTGGTTCCTGTGATACATTTCTGA
AMPL1420979	CTAGAGGTTCTGGGTTTTCTGAAAG	TATCCTTAATGGTGTTCTCTCTCTC
AMPL1420980	ATACAATAGCATTGATTCACCTTGT	ATGCGAACCTATGTCTAAATTCACT
AMPL1420981	TCAGTGAAGCAGGGAACAATCACTA	GGTTGTTGTAGTAAGAGCTTTGTTT
AMPL253545	GCTAATGTAGATTTTAAGGGAATTG	GTTTGTTACATGGGAATTGCGTGAT
AMPL253540	AGTGTTAGTTTTAATGGTTATGGCT	AGGAATGAAAACAGACCAAGGGCTA
AMPL253546	TGAGTTATGCGTACCTATAATTCTA	GGAACATTAAAGCTGGAAGGAGCTT
AMPL253552	GGTCAGACTCTAGATGTTTGTTCTT	AGTTATAAGGGCAATTAGGATCACA
AMPL253543	GGATGGTACGTTATCTCATTGTGTG	TTGGCAGTTGAGTGGAAAATACAGA
AMPL253551	TTGCTTCGGGTCAATATGAGTTTTT	AGAATAGTCAGTCCATAGGTCTAGG
AMPL253539	AGGCTTAACTACAGCTGTTCACTAT	TCTGTCTTTAAGAGTAGCTCTGAGA
AMPL253557	AAAGTTTGGCCTTACCTTCTCCACT	ATTACATTCATTTGCAGTTGCTGCA
AMPL253556	GCATAAACACTGTGAATACTGAAGG	TGACAGTGGGAATAATTCTCTTGTC
AMPL253550	AAAGGTGTCATAAATGACCTAGGAA	ATCACCATCGGAGGCAAAAACAAAG
AMPL253548	GTTTGCACATCACCCTGAGGAAATT	CTGTAAGGACACTGTTCTTCAGTGA
AMPL253547	AAGTACTAACTTCATTCCAGAACAA	TACCGCTAACTGGCAGTATTGTCAT
AMPL115721	CTGGTACATAGAAGGATGCAATAAA	GTTTTATATCTCACAACCTGGAAGC
AMPL253553	CCAACAGTAAAGTGAAATACTTTGA	AGATCAGACAAAACATAGATTTGCT
AMPL253554	GTGATACGATATTCATGCAGATACC	TGATGGATGGTTTTGGTAAAAATTT
AMPL253549	GCTCTAAACTAGTGGTCATATGCCA	CAAGTTGACTTTTCACATTCATACA
AMPL253544	TTCTGTTGATCAGGTGGATTTTATT	TAGTGGAACTGACCCAGCTACTCGG
AMPL115703	TTAATTACCAGATTGAAAGTCCCTT	TGATCTTTTTCATGAAAATAAGCCC
AMPL253541	AGATGCAGTCAGAGGAAATGATACG	ACTGTCAAAGCTTCTGCTTTCAGAT
AMPL253542	TTGAACTAGTGGTTAGGAAAACTGT	CGATGAAATTTTAAGACTTGCCCAG
AMPL1420984	ATGAGACTTCTAAATTTGCCAGTCT	GCAGTAAATAAGGAACCTGTTTATG
AMPL1420982	ACCGTGTTGTTTTCTTTTCACGGAT	TTTTCAGTCATCCTGCTGGTGCTTA
AMPL1420983	ACCCACCTGACAGAAGGGAAAATCG	CGAAAAGAAGACAGCCTTGGGTCGC
AMPL201372	GGCCACGGCTTCTTTTCTCCGTTAG	GAAGTGACGACAGTTCCGAAGGCGA
AMPL201373	GGAGGTTATTGGCCAAGTCCGCTAA	ACTTCTGGAGGAAGAAGCAACGCCA
AMPL193505	TCAGGATCTCGAATCAGGCGCTTAA	TAGAGGCACATTCTGGTCAAAGGCA
AMPL193467	ATTCATATGCAAGGCATAATGATAT	TCATGTTCTAGTTGACGGCAGCAGA
AMPL193469	CAAACTTATGCAACAGTTAAGTCCT	GCAACTTTTAAACCACCTTGCTTAG
AMPL193449	TGCTTTCTTTGGTGAAGTTTCATTT	ACAAAAATAAGACACTTGGAATGGC
AMPL193485	ACTGAGTCTAAAACATGGTCTTGCA	TCCCATACAGAAAAAGGATTTTAAA
AMPL193453	ACCTGAAGAGCTCTTTTCTTGTCTG	TGAGTACTGCCATACTAATGTACAA
AMPL193454	AGTCATATCCTCCTAAAGAACACTA	ACAAGCTCAACTGGGTATAACAGGG
AMPL193506	ATAGGCTTTTTGTGAGAACACAGGT	AAGCTGCATCAGCGTTAGAAGACAC
AMPL193468	CTTAGTTCTGGTTGAGATGAAAGGA	TGGAACAGCACGATCTCGTATCACC
AMPL193161	CTCTTGTCTTGACACAATGCAACTT	TCCTAAATTGGCAGGAAAATTGTCA
AMPL193163	TGATCAGGGATATGTGAGTGTGTGT	CGTAACACATATGGTGTACGTTCCC
AMPL193475	GTGGAGAGAGCCTGATAAAACAAAC	TATCTATCAGGACAGTGCCTGGCAC
AMPL193481	TTAAGATGCAGCTACTACCCAGCTA	GGACAATTTAAGGAGGACAACCATT
AMPL193492	GCTTTATACTGTAAACTACTGGATT	CCAGAACAAAAATAAATCACTCTGT
AMPL193479	CCACTCAGAAAATCTAGCTTTAACT	TTTTGACAAGCTATACACATTTCAG
AMPL193486	GTATGGGTATGGTATGTGTGTTGCT	AGAGTAGGACCTAGAAACAACATTC
AMPL193461	TAGCAATGGACTTCACCTCATCAAA	TCAGTAGCACCCTTAGCAAAAAGAA
AMPL193462	GCTAGATAATGATTACCACAAGCTA	TCACCAGATGTGGAATCAAAACCTT
AMPL193460	GAAGGTGTCAACCAATAAACTTCTT	ACTCTACCTTTTTCTTCTATGCAGC
AMPL193480	TAATGCTGACAAGTAAAATACCATT	TTTGTTCATAGACCAGTGAACTTCC
AMPL193472	CAGGAAGAACAGGATAGAAAGACTG	AATTAGATACTCGGCTACAATAAAT
AMPL193497	ACAAAGGGAGAAGCTACGTAATGAC	AAAATCCAACTGAAAAACAGCAGGA
AMPL193498	TGCTCTGTAATTGATTACAAAGGTT	ACAAGAAGGCCTAATATAGGGAGAA
AMPL193503	TGCTGAGTAATACGCAAATCCTTAA	ACTACAAGATGGGTACTGAGGTTAA
AMPL1149	GCCTACGTATATATTTTTAATCACA	TCAGGAAAAGGATCTAAAAGCTTAA
AMPL193452	AACTAAATGATGAGAGTCTGAACTA	GCCATTTATAGACACTGACTCATGT
AMPL193482	TACTACAGGCAACAGAAAACATACA	AAAGGTTTTAAGTCATGAGTAGCTC
AMPL193476	GTGAAGTATCATTCTCCATGAATGT	ATTAACCTTAAGGCCGAGTGCGGTG
AMPL193504	TCATGACCCACAGCAAACAGAACTG	CTTGATGAATGTTAACCTCCACTGA
AMPL193499	GTATCACAGCACTCTTTAGATAAAC	TCATTGTACCTTTCCTACACAAAAT
AMPL193450	GACAAAAGTCAGTTTTCACCTAGAA	AGTCTAGGCCATTACCATAATGAAA
AMPL193451	CTTTCTCTAGAACTGAGTTTACAGT	ATCCACCCAATAGTTTGAGTAAAAA

AMPL193457	TGAATATGGATTACTGCAAGGAGAT	TTCTGGTGGGCAGTACCTAACACAA
AMPL193491	GGAACAGGAGGCAAAATAAATCTTT	ACAGAAAATTACAAATTCCCAAAAA
AMPL193484	GCTTAGTCCAGTAAGTAAATTCAGT	AAGGTGGCAAACAAAACAGAATAAA
AMPL193113	GTGAAAATAGCTTTGGGTTTTACAC	ACAGTTCAAACAACACGAGTTTGAA
AMPL193288	TTATCTGGCTGTGTAAATATCCACC	AGCCATACAAACTATCTGGCTCCCC
AMPL193474	AGTTGTTGTTTAGAATGAGGAGAGA	TAAAATCCCAAAATATCAGCTGGGT
AMPL193343	TTATCTCTTCATCAATGCAAATCCT	ACATGTATAAATAGCTTCACCAAAG
AMPL193458	CACTATTGGTAACAGAAAAGCTGCA	ACCCTTGAACAACACAAATTTAAAT
AMPL193444	GTTGTCTATGTGTGACTGATCTGAA	AGAATGCCTCCATCTTTAGTCCTGC
AMPL193445	TTTTTCCCTCAGGCTTTCTGTTTTT	AGAGTTCATGTAAGCAAGAGAAATG
AMPL193489	TTTGCCCAGAATACTTGTGCTTCTT	AAGGACCTCTCTGTCTGCATGACAT
AMPL193490	TGATGGACTGATAGAATTGTGATAT	TCAGAGACTCCACAGCTAACTGAAT
AMPL193448	AGACTATACAGAGCTACAACAGTAA	AAAATGTTTTTCCTTGGTGGGTGGG
AMPL193493	GCTCATCACTACTTTCTCCATCATA	ACTATTGCAAACTTGTCCAACCCAC
AMPL193494	ACTCAGTTAACTGGTGAACATAAAA	CTTCTACTGCCTTCAAGAAAACACA
AMPL193483	GCTTATGTCTAAGTTTTTCAAGCAC	AGCCATGAACTTTAACAGAACTACA
AMPL193495	ATTGGTACATAAGAGGCAAAAATTT	TCTGAGTGTGACTGTGATCTTGATT
AMPL193496	TGAGGAATTAGAGAGAGAAAATGAG	TCTTCATTCCGTCTCTCTACCATTA
AMPL193488	TTTTAGATGGCATCTTAATAACACA	ACTGTCTAGATACTGCAGTGGGTAG
AMPL193459	GATTACGTTTGTGATTTTAAGCAGA	AACTACCCAACATTTAAACTTCTCA
AMPL193114	GGCAAAGGAAATATAATACTGAGAA	TGTGGTTCAGGCTCTGATTGAGAAA
AMPL193470	CGAAATTCTGCTTTAAATGACTGTA	TGGGTTATAACTGCTAAATCTGGCA
AMPL193471	GTTCTAGAAAATAATGCATTTCTAC	TTTCCATATTCTCCTGTGTGGTCCA
AMPL193500	GGCCTCCCAAAGCATTATGAATATG	AGTCTCCCAAATAATTAAATACCTA
AMPL193473	CTGACAGCTGTCAGCTTTAATAAGC	ACAAAGCTCCTTGGAGGAAACCAAT
AMPL193455	TGAATCAAACAAAAATAAAACCTGC	ACAGGATACATTACCTACAAGAAGC
AMPL193213	CAACTGTTTATGCTGAAAACAAAAA	CATAATTTCAATTAAGACCACTGAC
AMPL193112	CATGCCCAGCCCATGTAATTTTGAC	TGTGAGATCCACCTCAAATCCAAAT
AMPL193464	GAAAAGGCTAAGTGCACAATCTCCT	CATAATACTTGGCAGAGTGGGCTTT
AMPL193501	ATATCACTAAGGACAAAAACACAAA	AAGCCAGTCACATGGTAGTTGATGG
AMPL202650	AAATTCACTTGTCCACCAACACTGA	TGGATCATATAGAAGGACCTAAACA
AMPL433267	CGCCCAGCCCGAATGACCATTATTT	TCTTCCACTCCTTTCAGTTTCTCTT
AMPL433268	AGAGATCTGAAAAATGATGCATAAT	CACTGAGTGATCTTACTAGGAAAAA
AMPL433269	TTCAGGTATGGAAACCCTAAAGCCA	AACAATTTCTGAAAGAAGAAGCCCA
AMPL433270	GGGAATGAAAAACCAGAGAGGTGAA	TAAGAATGGGCTTGTGTGCAGCAAC
AMPL433271	GAAATCCTTACGGTCAAAAATGAGG	ATGTGAGCTGATGCTGTAGCTGTCC
AMPL321499	CGAGTCAGTAGTTTTACTTTCAGGT	ATCCTTACGGTCAAAAATGAGGGGA
AMPL433273	TGAACAGTTCTCCAGAAGTCAAACC	TGATGTTTATCTTATATGATGACAG
AMPL433274	AAGGCTAAAGAGTAGATTAATGATG	AGTCCAAAAAGTTCTGGAGATTGGT
AMPL433275	TTGGCAGTTTTCTATCTTAGTCATT	TCTCTGGATGCAACAACTAGCAAGA
AMPL433276	TCTTGCCTTGGACAACTTATTTCTC	TTCCAGCTTGCCAAAACCTTTTTTT
AMPL433277	CCTAAGTGCATACATTTTAGGCATA	TTCTCCAGCAGTGTATTGGGATAGT
AMPL433278	GCACACAGAACACACAAATATTTGA	CCAAGAGTTTGTGAACCTACTGATT
AMPL424	GGCCGGAGTAAGCTGACAAAAACCG	TTCTCGCTGACCCTTAAGAGTCCCA
AMPL425	GGGCAAGGACCTTTCTCTCAGGCAT	CTAGTTTCAGAAGCTCGCGCCACAA
AMPL476	ACATAAGGAACAGTTTATGGTTCTA	AACAGCTTACTGTGAGGATGGGAAC
AMPL418	CACAGGTTTGCCTAAATTCCTAGTT	GACAGACAAATTTGTGACCCAGAAC
AMPL442	GATCTTCTACCAGGCTCTTAGCCAA	TGTAAGAAGAGGTTTTTCAGTTTGC
AMPL429	AAACTTTGGTGTATGAAACAAACTC	ATCATCAAGTGATGTTTAAAATGCC
AMPL475	ACTTAACGTTTACTGAATTGCCTGT	ACAGGACTAAAATAAAACAAAGCAA
AMPL426	ATGACAATTATCAACCTCATCTGCT	GCAAAGGTATAACGCTATTGTCAAA
AMPL439	TATAGGACCAGGTTTAGAGACTTTC	GAAAGGTCAATCAACACAGTGAATA
AMPL473	CATGACTTGCAGCTTCTCTTTGATT	AGGAAAGGAAAATGGAAATGGAGAA
AMPL474	GTGCCAATTAAAAAGAGTAGTTCAG	TGTCTGTCACAGAAGCGATAAATCT
AMPL412	ATGTGGTCTTTGCAGCTATTTACTT	AGCAGTTCTACACAATTAAAACTCA
AMPL413	CTTCCACTCTCAAAGGGCTTCTGAT	CCTGATGTTTTTCCAAATCCTGTTA
AMPL414	ACTGTTTCCTCATTTAATGGCTTCT	ATCTAATGGATCAGTATCATTTGGT
AMPL415	CTTTCAGAGGCTTCAGTTTCTTTTT	AGGCTAGAAATACGTGGCAAAGAAT
AMPL416	GCAAATGTAAGTGGTGCTTCAAAAG	TCAGTCATATGACCTGAAAAACTTG
AMPL417	ACAATCATGTATACAGATGATGCCT	AACTGGGCTGAACAGTTAATTAGTT
AMPL450	CTTTTTGGATCATTTTCACACTGTC	AAAGGTAGTGTTTGGGCACAGTGGC
AMPL451	CATTTTGTATGATTCTTTGCCTCTA	CATGACAGAGAATCAGCTTCTGGGG
AMPL452	TCATTAGCTACTTGGAAGACAAAAT	TAGGTTTGACAGAACATCCTTGGAA
AMPL453	CTAGAGTCATTTTTATATGCTGCTT	TCTGAGAAAAGTTCTTCAGAGTCTG
AMPL454	ACTGAGGCTTGCTCAGTTTCTTTTG	TGTTCTCCTCTGCAAGAACATAAAC
AMPL455	AAGTGGTCTTTAAGATAGTCATCTG	CCAAGGTATTTACAATTTCAACACA
AMPL456	TTGGATGTACCTCTGCAGAAGTTTC	CTTCAAATGTACTCTTCTGCAATAT

AMPL457	GTAAATAGCAAGTCCGTTTCATCTT	TCTCAATATCACTAAACAGTTTCAC
AMPL458	CTTGAGCTTTCGCAACTTCCAAAAA	ACAGTATCATTTTTACTTGAATCAC
AMPL459	TGGGACACTTTCTTTCAGTATTTTG	ACAAATCTTCTTTAATCTGAGTGTT
AMPL460	CTTTGCCCATTGATGGCTAAAACTG	GTCTGTTTCCTCATAACTTAGAATG
AMPL461	TGAATTTTCAATGACTGAATAAGGG	AGTACCTTGCTCTTTTTCATCAAAA
AMPL462	AGCTGTTAGACATGCTACTGTTACT	GTAACAAGTTGCAGGACTTTTTGCT
AMPL463	CCCTACCTCAAAATTATTACTATTA	CTTGTTTTTCGGAGAGATGATTTTT
AMPL464	TCCAGACATATTTTGGTTATGTTGT	TTTAATGGCTGCATTTTTATTTTTG
AMPL465	AGACTTGCTTGGTACTATCTTCTAT	ACTCTGAATGTCAGCAAAAACCTTA
AMPL466	CTCTTATCAACACGAGGAAGTATTT	CTTGTCTTGCGTTTTGTAATGAAGC
AMPL467	GAACATCAGAAAAAGTTTCAGTTTT	TGTCTAGACGTAGGTGAATAGTGAA
AMPL92543	ACCAAGTCTACTGAATAAACACTTT	TTTTTACGTTTTTAGGTGAAGCCTG
AMPL422	TTGAAGCCTTTAAGGATTTTTCTTG	TGCTACCAGATACCACAGATAAGCT
AMPL95	AAAGAGGTCCTTGATTAGGCACAGT	AATAAGTTTCTTTTGATTGAGGGTT
AMPL444	CGGGAAGTGTTAACTTCTTAACGTT	TGATTCACAAATTGATAATGACTAT
AMPL92544	CACACTGTTCAACTCTGTGAAAATG	TCATTTGCTACATGGTGATACTATT
AMPL92545	AACCATCAGGACATTATTTAACAAC	TAAAAGGTGGAACAAAGACTTTGGT
AMPL91517	AAAATTACACTCTGTCATAAAAGCC	TGGCATTCTGAAGACTTGTAATTAA
AMPL477	CTCTGCATTTTTGCTGTTAATTTTT	TGCCATAGGAAGGTACTGTGAGGAT
AMPL91528	AGGGATGAGGGAATACATAAAAGTT	AGAAACGCCATACGTATACAGCTAC
AMPL471	CTACACAGAAACCTTAACCATACTG	ACCACATAGGATGATACTGAATTCA
AMPL92541	TAACAGCATACCACCCATCTGTAAG	CCACTGTTTAAACAAGTCACTGAAT
AMPL92542	CAAAAACTTTAACTGTCTGAAGAAT	CCACTTTTTGGGTATCTGCACTACT
AMPL427	AAAGAATCCAAGTTTGGTATACCAG	GCTACAAGTTCTTAATGCTATTTAA
AMPL428	AGGTGTGGTGGCTCAGCTACTTGAG	AGTACTGTTAGCAGAAATCTGGACA
AMPL359	CCTCTCTTTCATTGCGAAATATGTA	GTTAATTGTATCAGGCCAGGCACAG
AMPL472	GCTATTCTTTGTCTAACACCAAAAA	ATGTCTTCTCCATCCACTGTAATAA
AMPL430	AGCATTTCAACATACTCCTTCCTGT	ACACCCAAAGAAGGGAGATTTAAAG
AMPL446	CATTTGCCTGTGATTATTCAAGGCT	ACCAACTACATTACAGATGGCTAAT
AMPL448	AGTAGTGGATTTTGCTTCTCTGATA	TTCACTGAAATAACCCTGTGACCAT
AMPL419	AGATTCCATAAACTAACAAGCACTT	GGCATTAGTAGTGGATTTTGCTTCT
AMPL438	TCACTAGTTAGCTAGCAAATTTGCC	TACCGGTAGTTGTTGATACTGAGTT
AMPL423	GTCTATCCAAAACTTTATTGCCAGT	TGTGCAAGATTTCTGGAGATAAATT
AMPL92546	TTCCTTGATACTGGACTGTCAAAAT	TTCGTCTGACAAATAGACGAAAGGG
AMPL440	AACAGAATATACGATGGCCTCCATA	TGTCAAACATGCCGATGCAAAATTA
AMPL431	CAAATGGGACTAACAGGTGGAGGTA	AGGTTAGGTGATTTCAGTTCACATT
AMPL432	TCAGAGATGTAGTACAACGTCGTTT	CTCAAGAAATCCAAGGCTCTTCTCT
AMPL433	AGCAAAAGTATACCAATACGGAATC	AATCTTCTGAACTGGTGGGAGCAGT
AMPL434	CTCATTCCATTTAATGACACAATCT	TTTTTGCCCGATACACAAACGCTGA
AMPL435	TATTTCATAGTGAGTTACCTCTAAA	AGCTCAGCCAAAGATGTTTTTCTTG
AMPL270716	CCACTCTTCATATTCATCCTGACTT	TCTGCATCAAAATAACTGTACTAAG
AMPL1139776	TTGTGGTAAGTTTAAAAGGGCATAG	GGAACTATAGCAGAACCAACATATT
AMPL1139772	GATTTTTGTAATTTCCCACATTTGT	CATGCATCTTTCATGCCAGTGCTGC
AMPL1139773	AACATTGGTCTTTTGTGAAATGGTC	TAAAGGTGTACATAAAGCTTCAAAT
AMPL89490	TTGGGAACATGGTTTTGACCTTTTT	CTCATTTGCTGGCTGGACAAAAAGA
AMPL91681	GTCATTGCTTTAATTGTTTGGTTTT	TGTGTCACTTAGAAGAATGGAAAAC
AMPL91680	GTTTTTTCCCTGGTCACCTCCTAAG	TGAAAATAAGTGTTGGTACAGTGAT
AMPL91679	GTTTAGTATACACGTTTTCTGGGTT	ACAGGGTTGTTGTGAAGATTAAATA
AMPL91687	TAAAAGGTTACTCCTCACATCACCC	ACACTAGGTTATGACATAGAAGCAG
AMPL91688	TTCAAATGATGAAAATTATCCTTGT	AACCTAGGCTTGAGTCTTGAAAGAA
AMPL91696	TCACTTTGCATACTTATGCTTTGCA	AAGGCAGAACAGAGATGAGGTTTTT
AMPL91685	GTGAAACAGTTTGCATTTGGAGCTT	CAGATTCAGAAAAACAGATTTGGAT
AMPL91682	CTACAGAAGTTGCTGGACGAACTTG	ATTGGGCCTTCCACGTTTAAGAATG
AMPL91683	TCTTAAAGAGGGAAGCTGTATTTTT	AACTTGCTCATTTGAAAGACTCAGT
AMPL91684	TCTGTTTTGTTGGGTTTTGTTACTA	GAGTCCTAAACGCATGGATACAGAA
AMPL91690	AACCAAGTTCAAGAACCTCTCAGAA	CTTTCTTTTCTAAAAAGTGACAGGG
AMPL91691	AGTGAGATTCTAAGTCAACCTAAGA	TAACTAAAGTCAGCTCTCCCGCTGG
AMPL91692	GAACTTACTACTCACGACCTAAAAA	GAAGCAACCTCTCCTCTTTCTGCAG
AMPL91693	ACAGGAAGAAATCAGTAGCAAAAAT	ACTTCACCTGTAAGTTTGGAGGCAC
AMPL91694	GCCTGAGTCCTTTAACCCTGGAGAT	ACCAGTAACTGAAATAAGAACTCAC
AMPL91695	AATGAAATGTCACTGATTCTTTCTT	ATTACCTATACAAAGAACAGATGAC
AMPL91686	AGGTAAGTGAATCGTATTCTCAAAT	TGTGAAGATAATGACTAGCAATAGA
AMPL91678	CAAAAGTAGATTGTTATGGACCAGT	TTGTCTCCTCTAGCGTGCCCAAAGA
AMPL1139774	CGACGGCTGCTCTTTTCGTTCTGTC	TGTATCGACACCCTTGCTCCTCCCC
AMPL1139775	GGTGGCCCACTGGGACTCATCGACA	TGTGAGGAGAAGGAAAAGGTGCCGG
AMPL1140909	CTGGAGTCTGAACTGACTTCCGCAA	ACACTTGGCTGAGTTCTTTTGTTTT
AMPL1140910	CCCGAACTTTCTTGGAAGAAGGGAA	TTTGCAGTTCCGACGCCACTGAGAG

AMPL193106	GAACGTGTAGCTCTCGGCGTCAAAG	AGTCTCCTTTCTCATTTTATTGGAG
AMPL193107	CCCCAAATCTGCGTAAATTCCAAGG	GAAAGGTAGATGGAACCGGGTGACT
AMPL193101	TACCAAGGCTGAGAAACCTGGATTA	TCCTTCTCCAAAGAGCGAACTTGTG
AMPL193094	GAAGGATCCCAACACTGGGTCTTTT	TTCTGTGGGCAGTGTACAGTTTGAA
AMPL193100	TCCTCATGTGTTCAGAGCCTCTGAT	AATCCCTCCCAGAGAAACAGAGAAC
AMPL193110	AACCTTTGGGCTTGGACAACACTTT	TGACTTTTCTCACACATGTGCTGCT
AMPL193104	ACTGACAACTGGCCTAGCAGGATTT	TTCTGCCCTGAGGGATAAAGGAGGG
AMPL193103	CTCTAGTCTGGAGAGAGTTTTCAAA	TTAACCATGTGCTTCATGCCAGCCT
AMPL1140908	GTCCACTACAATCTGGGAAAGTCAC	CAAGACTGCTAGCAGAGCTGAGTCT
AMPL193092	ACTGCTACGTGTAGAATGTACTGCT	TCAACAAGCAATTCACTCAAATTCT
AMPL193093	ATGACAAAATGCCATGAAGTACATA	AAATCCAAGCCCTAGAGAGCAGAAA
AMPL193102	AAAAAGAATGAACTCTTCCCTCCAA	TTTTTATTTTGAAAACCAACAATAG
AMPL193108	TGTTCCAGTAGCAACTGGAGAACCT	TATTGCCTGCCTGTTGTTTCCCTAG
AMPL193109	AGTTGAGAAGGGAAATTGAAAGGTG	CACAGAAAATGTGGCAGCTTGGCAA
AMPL193098	TTTCCAGGAAATAAACCTCCTCCAT	AAAGCTCATTCCAGAATAAAATTGC
AMPL193099	CCAAAGTGATTCAGAGCTGTTTCAA	ACTAGCTGCCTTCAATACACTTGAA
AMPL193097	AGCTGAAAACATAGTAAAGGAAAGA	ATGCCTTCACTGCCAACTGTATGAT
AMPL1140901	CTCAAGGGAAGGGAGCTGAAAAACC	TTAGTGAAAGGAAGGGCACACCTGT
AMPL1140902	ATGCTAAAGTTGTTCTTCTGGAATA	ATTTCTGCATTTCCCAGCACATGGG
AMPL323542	AAGGCAGAGGGACACACCAGTGTAG	GTATCTTTTGGACATCACCACCATG
AMPL1140903	TGGGCGATGGAGCGAAACTGTCTCT	ACACACCAGTGTAGTAATGAGCAGA
AMPL1140904	CTTGGGCAAAGCAACTGAATTCAGG	TGGAGCGAAACTGTCTCTTAAAAAA
AMPL1140905	TCTTGCTTCAGAGCTAAATAAACAT	ATATTAAAACATGGAGGCTGGGCAC
AMPL1140906	TGGAATTGGGCAAATGTGTTCAGCT	CTTACTCAGAACAAGTCACTGGATA
AMPL1140907	AGTGACATTCTCTACACAGAACTAT	TTCTGTAGACATCATCAAAAGCAGG
AMPL323549	TCTTTTCTCTTGACTGGAAATGTGG	CTTGAACTGCCGAAAAATCAAGACA
AMPL328876	AACTTTTGCATGTTTTGTAGAGATG	AGTCTGCAATGAGTGTGGGCTGGGG
AMPL275183	TTATGCTCAGGGTCAATTTCTTTTT	CAACATCTTTTACATTCTGCAAGCA
AMPL275185	CCATATCTGTGAAATGCTGGCATTT	CCAAGACTTGTTTTATGCTCAGGGT
AMPL275186	TTTGAACCCTTGCTTGCAATAGGTG	ACAGAGTGCATTGTGAGGGTTAATG
AMPL328877	TTCAAAGCATTGGTCAGGGAAAAGG	AGGACTTCCATTTGCTTTGTCCCGG
AMPL91542	TTGCTTTTGTACCGTCATAAAGTCA	TTTTAGTCTTCCTCCGGTTGAATCC
AMPL92563	GTTGTAGCTAACTAACTTCAGAACA	TCTAAGGAACATCATAGCTTAGCCT
AMPL91545	AGGAAGAGAATCTCCGCAAGAAAGG	CAAAGACAATGGCTCCTGGTTGTAG
AMPL91541	GGACAAGGGTGGTTGGGAGTAGATG	TTTCCTTGCCTCTTTCCTAGCACTG
AMPL91543	ACTGGCCTCATCTTGGGCCTGTGTT	TACCTGGAGCTGGAGCTTAGGCTCC
AMPL91544	ATCTACAAGCAGTCACAGCACATGA	TTCTCCTGCTGCTTATTTGACCTCC
AMPL275179	TCTCCTTCCTCTTCCTACAGTACTC	ACAGAAACACTTTTCGACATAGTGT
AMPL267718	AAAGAAAAGCTCCTGAGGTGTAGAC	ACAAGATGTTTTGCCAACTGGCCAA
AMPL275190	TCCCAGCACTCTCAAAGAGGCCAAG	CTAGCTCGCTAGTGGGTTGCAGGAG
AMPL91540	ACGATATTGAACAATGGTTCACTGA	TTCACCCTTTGGCTTCCTGTCAGTG
AMPL328874	TTTCTGCTCTTGTCTTTCAGACTTC	CTTCTGTCCCTTCCCAGAAAACCTA
AMPL328875	GACCCAGGGTTGGAAGTGTCTCATG	GTTCTGGTAAGGACAAGGGTTGGGC
AMPL328878	CCCAACTCCATTTCCTTTGCTTCCT	ATTTTCAGCTCGGGAAAATCGCTGG
AMPL827588	TTTGCTTGCTGAAGGAAGAAAAAGT	CCAAAACAAATTGTTGCAAGGTCAG
AMPL827589	GAAGAGATTTCTAAAAGTCTGAGAT	ACCCATTATCCAGGACTGTTTATAG
AMPL574036	GAGGCAGGTATTAGAAATGAAAAGG	GAAACTAGAAGAGATTTCTAAAAGT
AMPL574037	TTCTCTAAAACCCTGTGTTCACAAA	ACTTGAAACCTGGGCATGGTGGCTC
AMPL827590	CTACTGTCCTGGCTACTAAATATTT	AGACCCTTCAATGGAAGGAGAGTGC
AMPL934428	TCCAGGAGAATGAATTGACACTAAT	CCCTTAAAGATTTTCTGCTTGAAGT
AMPL3006	ATGATGAAGTGACAGTTCCAGTAGT	AAAGTAATACATGAACTGTTTCCAT
AMPL2996	TAAGACCATTGTCCTTTGGAGCAGA	ACTCCATCTGTAAGGGATGGGTAAG
AMPL2994	CTGGGAAGTAGCAGCAGAAATCATC	TCTCCAAACAAAACAGCACCTAAAT
AMPL2993	GCGCCTGGCCTGAATGCCTTAAATA	TTCCATCCCAACCACATAAATAAGT
AMPL3020	CTGCTACAAGCCTTATTAAAGGGCT	CAAGAATTATGCAGGCCTGCACTGT
AMPL3022	TGTTTTCAGCCTCTGATTCTGTCAC	TGAGTCTGAGTTACCAAAAGGTCTT
AMPL2999	AATAGTTCCAGGACACGTGTAGAAC	ATAAAACCACATGAGGCGAGGCACG
AMPL2990	CAGAATTGACAGCTTCAACAGAAAG	AGGACTTTCGAATTGGAATTTTCCT
AMPL2991	TGTAATTCAACATTCATCGTTGTGT	GAATGTCCATGGTGGTGTCTGGCCT
AMPL92530	TTACTTGCCAAGGCAAGATCTAGGT	GTATTATTTCAGGCCAGGCATAGTG
AMPL92531	ATGATTTGTCCTTTCACAATTGGTG	TGCTGTATTGGAACAAACACTTTGA
AMPL2998	AATTTGTGTATCATAGATTGATGCT	TGCATACAGGCATTTATACACTTTT
AMPL2989	CTTAACTTGTTTACAGCGATGCCAA	TGTATCTGAGCCTTCCCTCTGCCTT
AMPL3004	AGGTGATTTCAATTCCTGTGCTAAA	AGAGTAAGGACCTAGCTCCAACATT
AMPL3007	CCAAAGCAAGGAATTTAATCATTTT	TTGTGGTTCATTGTCTCCTTAAATT
AMPL3008	ACAAAATGTTCTGCTAGCTTGTTTT	GCTTCTTAAACTTGAAACATGTTCC
AMPL3009	AAAGGAGAGCTTAGCAGGAGTCCTA	AGTGAATTGGAAGACTTGACTGCAA

AMPL3010	ATTGAATGCTATGCTTAGATTAGGG	CATACACATTTGGCTCAGGGTTACC
AMPL3011	TTTTCCCATCAAGTCATTTGTTAAA	TGAGGTCTATAAACAAAGTCTTCCT
AMPL3012	CCTTAAAGAAACAAAGTCCAAAAGT	GAAAAATCTGCTAGAGGAAAACTTT
AMPL3013	TTAGCACTCTAGGGAAGGCAAAAAC	GTGAACAAAAGGAAGAAAATCAAGG
AMPL3014	AAATGAACAGACAAGTAAAAGACAT	AATGTGTGAGTCAGTGTGCAGCATT
AMPL3015	GTATAAGCAATATGGAACTCGAATT	TTTCCCAGAGCTGAAGTTAACAAAT
AMPL3016	ACTGAGCCACAGATAATACAAGAGC	ATTCAAAAGCACCTAAAAAGAATAG
AMPL3017	TAGGTTCTGATGACTCACATGATGG	AAATTAAAGCGTAAAAGGAGACCTA
AMPL3018	TGAATTCTGTAATAAAAGCAAACAG	CAAATGCCAAAGTAGCTGATGTATT
AMPL3019	CCAAGGTGTATGAAGTATGTATTTT	AAGGAGCCAACATAACAGATGGGCT
AMPL3003	TAAAGGAGAGAGCAGCTTTCACTAA	GTTAGAGTATCATTCTCTGTCAAAT
AMPL3021	TAACCACTTCTCTGTATTACATACT	TGTTATATAGGTAAACACACGACTT
AMPL3023	TTTCCAGGCATCATACATGTTAGCT	GGAAGTGAATTTTATGAGCCCTATG
AMPL3000	GCCAAAAGACTTCTACAGAGTGAAC	ACCATTTTCATTTATTCTGGCAACA
AMPL92532	GGGTTTCTCTTGGTTTCTTTGATTA	TCCTTGGTAAAACCATTTGTTTTCT
AMPL3002	GAGGTTTTCTACTGTTGCTGCATCT	ATTACTCCCACTGGTCTCACACCTT
AMPL2988	TTAAATGGCTCTTAAGGGCAGTTGT	CCTGAAAGTTTTATGGGACATCTGC
AMPL2997	CTGACACAGCAGACATTTAATAAAT	TCCAACTCCATTTCATTCTTTCTCA
AMPL2987	TTGTCATTAGTTCTTTGGTTTGTAT	AAGAAAAGACATGTCTAGTAAGATT
AMPL827587	TGCTCTGGGTAAAGGTAGTAGAGTC	TAAAAACGTCGGCTGGTCATGAGGT
AMPL574545	TGTCCCTCCCATCCTCTGATTGTAC	ATTTCCGAAGCTGACAGATGGGTAT
AMPL92975	TCCGTCTCTTTCCTTTTACGTCATC	ATTCTGAGAGGCTGCTGCTTAGCGG
AMPL193549	CAGGAGTCGTTACCTTTGCTAAGCT	CCAAAATCCCATTTAGAGATTCCTC
AMPL193550	GAGGAAGCAGAATCTAACGGAGACT	ACCTCTAGGAGTTCCTCAGCAGTCT
AMPL1338720	ATTTCTGTTGTTTTCATTAAGGGCA	TAGGAGTGGAGAAACATAAAAATGC
AMPL1338721	CCTTATGACTGCAGTACAATTTGCA	AGAATATCATCTAGTGCTGAACAGA
AMPL193556	AGGTCTCAGATGGGCACAAATGCAA	GGTAGTGTTTTTGAACTCCAGATAA
AMPL193548	TTGATTTTATGCTACTGTACTGGTT	TGTATTGGCAATTGTAGAAAAACAA
AMPL193554	AACGCTATTTTGACATTTCTAGACA	ATGCTCTCTCTCTCCAAGAGAGCAG
AMPL115344	AGCGTATAGTACTCAATGAGAATCA	ATCAAGTAGACCAGTCTGGCCAAGA
AMPL115331	CTAGCTCTAAGAAACCTGTAAGTGT	TCTCAGAACGCCTCTGATCAAATAT
AMPL193555	TGTTCTAAGCATCAAAAGCTGTCCT	AAATACACCCTTCAAATTACCCGTA
AMPL516562	TGTTTGCAATGAACATGCAGAAGTA	TACTTTATTCTAGGGCCAGGCGCAG
AMPL1338722	TTTAGAAAAACTTTCACAGAATCAT	TCTAAATCCCTGAGGCTGAAAGATA
AMPL1338725	ACAGGCTGCAAACCAGGAAAACTAC	TCATGAAATCAAAGCACCTGTTGTC
AMPL1338726	TGGCACATTGCAAGTTATTCATTAT	AGGGCTTTATTCTTGGAATAAAGGT
AMPL1338727	CTTTCCAAAATTTAATTCTCCCTGA	TGAATCATATATCTGGAGTGCACCC
AMPL1338728	CTAGCTAGATATGTTTTTGTTTGTT	TTCCAAAATTTAATTCTCCCTGAAG
AMPL1338729	GGACTTTTAAAATTACCTTTAGGGC	AGAATTGGTAAACTACATGAGAAAA
AMPL1338730	CTTATGAGACCTGATGATTTTATAA	AAATTACCTTTAGGGCCAGGCGCGG
AMPL1338731	TGAGAATCTATAAAGGAATGTCTGT	TGCTTCACTCTCATTCTTCTCTCCT
AMPL1338732	ATAGTAGGGTAGTGAAACGAATAAA	GTCGATTTTCATTTGCTTTTGTTCC
AMPL1338733	GTCTTTGCTTCCATTTTGAGACATT	AGACAAGAGACTTGGGCTCTACACC
AMPL1338734	TCTCTGACATCATTCCAAAAATACA	AGCCTGTTTCTATAGCATTTGATGT
AMPL1338735	ATTTATAGCTGCAGCTAAAATATGT	TTTTTAAGAAACATCTGTTATTCAC
AMPL1338736	AAACATGTCATAAACAAATTGACGT	ACAGTAATCCTTTCTGTGAATTTTG
AMPL1338737	TAGATTTTACCTCAAATCAGTCCAA	TTATTTAGCTTCACCATCATTGTGG
AMPL1338738	CTGCCCTTCTCTGCCTCCCTAAGTT	AGATCACCATTTGTCTTGAAAGGTT
AMPL193671	ACCTAGCTGAAACTGATAGAGGAAA	GTCATCATGCTTATGTTAAACTCTG
AMPL193672	GAATCCTCAAATCTGACAGTAAACA	ACAATTCAGATTGCATTTTAGCTAA
AMPL193673	TATGTTAGCTAGGAGCAGAAAGTTA	TCATGCCCTCAATCAGAAACCATTA
AMPL193667	TCACCAGAAAATTTTGTGGAAGATG	CTAACTGGCTTTGCCACTTACTAGC
AMPL193668	AAAAAGCAAGTGTATTACCTTAGCA	TGTGTCCAGGAACTACAGTGTCCTA
AMPL193662	GGATAGCTTCTGTAACTTTTCTGAT	ACATGAGCTCTACTATCTTATTAAG
AMPL193669	GCCGTCAGTGGTATGAAATTCAAGC	TGAATAGCACACAAAGATGCTCAGT
AMPL193670	TTAGGTAATTTAAGGAATGTGAAGC	TAAACCAGGCCCTTGGTAGGTAAGT
AMPL193648	CATAACAATAGGAATTCCTTTTCCA	ACTGTTAGGGTAATCTGTGATAAGT
AMPL193649	GCTACCCTTTTGCATATAAATGTAA	TCTACAGGTAGGCCATCAAAACCTT
AMPL193663	TATGACGCAATCAAATACAAAGGAG	AGCCAAATGAGACCTATATTCTGAT
AMPL115910	TTAAGTTTGTTTAGCTAGACACACA	TGGAAATTATTTCATGGTCGGTGAA
AMPL193658	CTAATTTCCATGCCTTTTTCAGGCA	TTCCTAGGGTAGAGGCAACAAGGTA
AMPL193652	GTTGTTTTCTTTTAGGCCTTTTCAG	ACTTGGGATGCAAAAGGCTGTATTT
AMPL193674	AGCTCTTTCAAATGAGTTTTAATGT	ACCAGCATTTTAATAAATGTGGTTG
AMPL193653	AATTTCAATGCATGTTTTGTTGGGT	TTGGGTGAGTAAATAGGAGAATTGA
AMPL193657	CGCATGACAATAATGATGATTCTCT	TCCAGATTAAATCTAGTGAGTAACC
AMPL193650	GAGCTGTGTGCTGTAGCCTCATTAA	GGATATTGAACCAGAAACTAAACGT
AMPL193651	TTGTTAAATCTATTTTGCCAAATGT	TGTCTATTAGTGTATTTGTGCTGCC

AMPL193666	GTTCCTCAAATTCTGAGATAAGCTT	TCCTACTCATGGAGATGTAAATGTT
AMPL193664	TCGGTAACTTCAACAGAAATGAGAA	ATCCTGCTACCAAAAATAGTCAAAC
AMPL193665	CCATGTGAGGTTTGATAACGTTTTT	TGCTAGATGGGAAAAACGTGAGTAA
AMPL193660	AAATACATAGAGCTGTTTTGGCCTT	TCTCTGTTACTAATGTGTGAGATGG
AMPL193659	TAAGCCTTACATCCTGGTTCCAGTT	TTGGGTCATGTGACAGCCATAAATT
AMPL193654	AATGGCACTTCATCAACTTGTCAAG	AAGGCAAGGCAAAATAAATGATTTT
AMPL193655	TGTAGAGAATGATTGATGTTTATTT	TTTGCCTGGGTCAGTTGATATCTGT
AMPL193656	TCACTTTTCCTTGCTGTTCTATTTA	TATCCAGACTGGTTCAGCCATGCTG
AMPL193661	GTTTTGGTTCTCTGGTTTTCTTTGT	TGATTGCAGCTAAACTGTATATATC
AMPL1338723	TTATTTGCTCTCAGAAGTCGGTTTC	TTGACACTATTGTTGCCACAGCCGG
AMPL1338724	ACTTCCATTGGATGCCGAAGTTCTC	TTCAGTGAATCGGAGCTCAGAGCGT
AMPL576709	CGCCGCCGCCATCTTGTTTACCTCC	CGTAGGTCACGGGTGCCAACGGCCT
AMPL576710	CCCTTCTCCAAAATTTTACAAAGAA	GGACAAGTATGAACACGGCCGAGCG
AMPL576711	CACCTCCTTCACCTTGCCGTAAGAG	CCAAAAGGAAGGGAAAAACCCTTCT
AMPL193005	CAGGGTGTTAAGAGGAAGTAAGGGA	CATCAGGTACTTGCCGATGAGCTTG
AMPL193011	AGCCATTGCCACAATGGCTGACTTC	ATCTCCCTGTGGAAGTGGCGTACCT
AMPL193012	GCAGTGTGGCCTCACGGAAAGGAGC	GTCCCAGGAGGAAGGAGCCTACGGG
AMPL193013	CGGCTTGATGTCCTTGTGCACAATG	AAAGTCTTGCTGGAACAGCAGGCCC
AMPL193002	GACCCCAGCCGACCAGATGTCCACC	CTGACAGAAGTACCTGCGGGCAGCA
AMPL96363	TGAGTTACATTTCCATAAAGTAAGC	TTGAAGCCGGAGAAGGTGTCCAGGC
AMPL193003	ACCCCCAACCCTACATTTCTGCACA	TTCACCACCTTCCACTAGTGGAGGC
AMPL193009	CTGCCGGATCTGCCGGATGGAGAAC	TAGGAAGTGCAGCAGGACAACCCTG
AMPL193010	GGACGGACGCATGTCCTCACTCAGA	TTCGTACTCAAGCATCCCTGAGGAG
AMPL193006	AAGTCCTGAGTGTAGATGATGTCAT	AGAAGCTCAGCCATGTCCTCTAGGA
AMPL3119	GGGCAGAAGCTGTCCTTGTTGCAGA	AAGAGGTCCTCGTCCTCGTCCGCGC
AMPL193007	CCGCCTCTGTGCCGTTCATACACAC	ACGTCTGTTGCACCTGCCATGGCCG
AMPL193008	CGTGACGGTCACCATGACTGACTAG	TGTCCATTGTGACTGGCCTCCTCTT
AMPL576695	GGGGCGTGGTCGGCACAGAAGCATG	TGTAACTCAGGCGGTGGCCCCCAAA
AMPL576696	GTAGTCTCCACATAAAGTGCATGGA	AGGAAGACGGAAGAGGTCAGCCTGG
AMPL576697	CAGCTCAAATCCACCTTTTATTTTT	CTGCGAAGTCAACAAGCCGCGTGCA
AMPL576698	GCGTTTCTCGTGTTTTTCTTTTCTG	AAAAAGAAAATGGAACCAACCAAAC
AMPL576699	GACCTCCGAGACCGTTTACCTCACT	TATTGACCGCACACTCCTGCTTGCT
AMPL1140898	GGAACTTTGATAATCTTAAAGAAGC	ATAATCGGTTCTTTAGTTGAATGGC
AMPL193569	GATGATTCTTGGACGGACATTTTTC	TAACTTGATTCGCTCCAGCACCCTT
AMPL193570	GCTGACTCCGTGATGATCTCAGACC	AGGATGGGTTACAACCTGTTTTATT
AMPL193563	GGGATTACAAGCCTAAGGCATTGTG	TATGTGCTGTGGTGGGAGTTTCTTC
AMPL193558	CTTCTTGGACTGGCAGACTATGTTA	ACAAATGTGTCTTGCTCTGTTGTCC
AMPL193573	TGGAGAATATGGTTGTGTGTATCAC	TCTGACCCTTTCACTGTTACCAATT
AMPL193571	ATAAAAATGTCAACTGTTTGCTTGT	GGCAAAAATGTCCTACTCCTTTCAC
AMPL193567	GTTGGCTAGATTCTGTCTGCCTTCT	TAGGCTTGCCTTTCTCATCCTGGGG
AMPL193561	TAAGTAGAGCTGGGTTTGGAACTCA	AATAAAGATAATATTGGCCAGGTGT
AMPL193562	TCTGCAAACAGGGACAATGGAAAGG	TGCTTAGATGGATGAATTTGTGAGT
AMPL193559	TTACAATATGTATTAGCCCACTAAT	GTGATTGGTGGTTTCTCATTTCTGA
AMPL321599	TCTTCCCTGGGCAAGAGAGTAAGAA	AGCTGATTTTCAAAAATACGGCGGG
AMPL193572	ACCCATGTATCTAGGAGAGCTGGTA	AAGATGAGTAAGTTGCTGGGCGTGG
AMPL193568	TTGATAATTCTGATTGCCTTCTTAG	CAAAATGTTCTCTATTTTAGGAAGT
AMPL193557	ACAGAGTCTGGCCATGTTACCCAAT	TCTCCTCTGATATCTGAAGACTAAG
AMPL193565	GCTCCTTAGAGACAGTGTCCACTCA	GTACATGTCACAGGATGGGAGCAGG
AMPL1140900	AGTCTTGTGCCTTGAAACTCACCTT	CTATTCCTGAGGACCAAGAACCTGA
AMPL1140899	ACCAGACTAATGTTGCTGATTGGCT	AGCTTCGCTTTGTCTGTAGTTTTGA
AMPL193009	CTGCCGGATCTGCCGGATGGAGAAC	TAGGAAGTGCAGCAGGACAACCCTG
AMPL193010	GGACGGACGCATGTCCTCACTCAGA	TTCGTACTCAAGCATCCCTGAGGAG
AMPL193006	AAGTCCTGAGTGTAGATGATGTCAT	AGAAGCTCAGCCATGTCCTCTAGGA
AMPL3119	GGGCAGAAGCTGTCCTTGTTGCAGA	AAGAGGTCCTCGTCCTCGTCCGCGC
AMPL193007	CCGCCTCTGTGCCGTTCATACACAC	ACGTCTGTTGCACCTGCCATGGCCG
AMPL193008	CGTGACGGTCACCATGACTGACTAG	TGTCCATTGTGACTGGCCTCCTCTT
AMPL576695	GGGGCGTGGTCGGCACAGAAGCATG	TGTAACTCAGGCGGTGGCCCCCAAA
AMPL576696	GTAGTCTCCACATAAAGTGCATGGA	AGGAAGACGGAAGAGGTCAGCCTGG
AMPL576697	CAGCTCAAATCCACCTTTTATTTTT	CTGCGAAGTCAACAAGCCGCGTGCA
AMPL576698	GCGTTTCTCGTGTTTTTCTTTTCTG	AAAAAGAAAATGGAACCAACCAAAC
AMPL576699	GACCTCCGAGACCGTTTACCTCACT	TATTGACCGCACACTCCTGCTTGCT
AMPL1140898	GGAACTTTGATAATCTTAAAGAAGC	ATAATCGGTTCTTTAGTTGAATGGC
AMPL193569	GATGATTCTTGGACGGACATTTTTC	TAACTTGATTCGCTCCAGCACCCTT
AMPL193570	GCTGACTCCGTGATGATCTCAGACC	AGGATGGGTTACAACCTGTTTTATT
AMPL193563	GGGATTACAAGCCTAAGGCATTGTG	TATGTGCTGTGGTGGGAGTTTCTTC
AMPL193558	CTTCTTGGACTGGCAGACTATGTTA	ACAAATGTGTCTTGCTCTGTTGTCC
AMPL193573	TGGAGAATATGGTTGTGTGTATCAC	TCTGACCCTTTCACTGTTACCAATT

AMPL193571	ATAAAAATGTCAACTGTTTGCTTGT	GGCAAAAATGTCCTACTCCTTTCAC
AMPL193567	GTTGGCTAGATTCTGTCTGCCTTCT	TAGGCTTGCCTTTCTCATCCTGGGG
AMPL193561	TAAGTAGAGCTGGGTTTGGAACTCA	AATAAAGATAATATTGGCCAGGTGT
AMPL193562	TCTGCAAACAGGGACAATGGAAAGG	TGCTTAGATGGATGAATTTGTGAGT
AMPL193559	TTACAATATGTATTAGCCCACTAAT	GTGATTGGTGGTTTCTCATTTCTGA
AMPL321599	TCTTCCCTGGGCAAGAGAGTAAGAA	AGCTGATTTTCAAAAATACGGCGGG
AMPL193572	ACCCATGTATCTAGGAGAGCTGGTA	AAGATGAGTAAGTTGCTGGGCGTGG
AMPL193568	TTGATAATTCTGATTGCCTTCTTAG	CAAAATGTTCTCTATTTTAGGAAGT
AMPL193557	ACAGAGTCTGGCCATGTTACCCAAT	TCTCCTCTGATATCTGAAGACTAAG
AMPL193565	GCTCCTTAGAGACAGTGTCCACTCA	GTACATGTCACAGGATGGGAGCAGG
AMPL1140900	AGTCTTGTGCCTTGAAACTCACCTT	CTATTCCTGAGGACCAAGAACCTGA
AMPL1140899	ACCAGACTAATGTTGCTGATTGGCT	AGCTTCGCTTTGTCTGTAGTTTTGA
